# Ecological engineering across a spatial gradient: Sociable weaver colonies facilitate animal associations with increasing environmental harshness

**DOI:** 10.1111/1365-2656.13688

**Published:** 2022-03-29

**Authors:** Anthony M. Lowney, Robert L. Thomson

**Affiliations:** ^1^ FitzPatrick Institute of African Ornithology, DST‐NRF Centre of Excellence University of Cape Town Rondebosch South Africa; ^2^ Animal and Agriculture Research Centre Hartpury University Gloucester UK

**Keywords:** arid environments, bird nests, ecosystem engineers, facilitation, niche construction, positive interactions

## Abstract

1. The spatial distribution of animals in a landscape depends mainly on the distribution of resources. Resource availability is often facilitated by other species and can positively influence local species diversity and affect community structure. Species that significantly change resource availability are often termed ecosystem engineers. Identifying these species is important, but predicting where they have large or small impacts is a key challenge that will enhance the usefulness of the ecosystem engineering concept.
2. In harsh and stressful environments, the stress gradient hypothesis predicts that community structure and function will be increasingly influenced by facilitative interactions.
3. To test this hypothesis, we investigate how the ecosystem engineering role and importance of sociable weavers *Philetairus socius* varies across a spatial gradient of harshness, for which aridity served as a proxy. These birds build large colonies that are home to hundreds of weavers and host a wide range of avian and non‐avian heterospecifics. We investigated the use of weaver colonies on multiple taxa (invertebrates, reptiles, birds and mammals) at multiple sites across a >1,000 km aridity gradient.
4. We show that sociable weaver colonies create localized biodiversity hotspots across their range. Furthermore, trees containing sociable weaver colonies maintained localized animal diversity at sites with lower rainfall, an effect not as pronounced at sites with higher rainfall.
5. Our results were consistent with predictions of the stress gradient hypothesis, and we provide one of the first tests of this hypothesis in terrestrial animal communities. Facilitation and amelioration by ecosystem engineers may mitigate some of the extreme impacts of environmental harshness.

## Graphical abstract

A demonstration of how sociable weaver colonies create localized biodiversity hotspots across their range. The results are consistent with predictions of the stress gradient hypothesis, and the authors provide one of the first tests of this hypothesis in terrestrial animal communities.

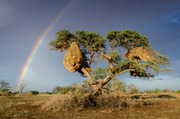

## 1 INTRODUCTION

The spatial distribution of animals within a landscape is largely determined by the availability of resources (Hunter et al., 2012; McIntyre & Wiens, 1999), which can be concentrated in specific locations (Parrish & Edelstein‐Keshet, 1999). Resource availability can be facilitated by other species, and this can positively affect local species diversity and impact community structure (Soliveres et al., 2015). Species that considerably alter resource availability in an environment are known as ecosystem engineers (Jones et al., 1994). This concept is not without criticism (Reichman & Seabloom, 2002), but it is agreed that significant value can be derived by identifying those ‘engineers’ that disproportionately influence resource availability and have the greatest abiotic and biotic impacts on their environment (Coggan et al., 2018; Crain & Bertness, 2006; Romero et al., 2015). However, the impacts of ecosystem engineers have mainly been carried out across small spatial scales, limiting our understanding of how spatial context may alter impacts (Coggan et al., 2018).

The stress gradient hypothesis (SGH) predicts that the significance of facilitative interactions will increase in communities in harsher environments (Bertness & Callaway, 1994). This is supported by empirical evidence demonstrating greater associative and positive between‐species impacts with increasing environmental stress, often facilitated by identified ecosystem engineers (He et al., 2013). To date, studies into the SGH have almost been exclusively tested in plant communities; however, recently, ecologists started to apply these ideas to animal communities (Dangles et al., 2018; García‐Navas et al., 2021; Lowney & Thomson, 2021). Moreover, studies across broad spatial scales are challenging to replicate but may demonstrate the importance of engineering species to different communities and in different contexts. Environmental conditions (i.e. aridity, altitude, salinity) will likely vary significantly across an engineering species' distribution (Coggan et al., 2016; Erpenbach et al., 2012). Therefore, monitoring an engineer's impact over large‐scale spatial ecological gradients would enable a greater understanding of how engineers may facilitate or mitigate condition in environments differing in harshness.

Species interactions are likely a key factor as communities respond to climate change (Alexander et al., 2015; Harrington et al., 1999; Suttle et al., 2007). In arid environments, climate change will predominantly cause increasing frequencies and duration of hot weather or drought events (Akoon et al., 2011; Meehl & Tebaldi, 2004), and a reduction in rainfall (Osman et al., 2017; Ouhamdouch & Bahir, 2017), conditions that will make these environments harsher to most species (Erasmus et al., 2002; Isaac, 2009). Altered species interactions due to an environment becoming too harsh may lead to a loss of certain species from communities, long before species‐specific temperature thresholds are reached.

Ecosystem engineers join the abiotic and trophic aspects of communities via their interaction networks (Sanders et al., 2014). Engineered structures that provide thermal refuges may be crucial under increasingly higher temperatures (Coggan et al., 2018), resulting in increased use of these structures. Burrowing by animals may provide these refuges but also alters the soil properties that directly influence local plant community composition (Bancroft et al., 2008; Whitford & Kay, 1999). Therefore, by influencing plant biomass they have the potential to provide resources in periods of low plant productivity. Bird nests have the potential to provide resources for many different species as they come in different shapes and forms, with large communal nests providing resources for species that gravitate towards these structures, and nests burrowed underground that alter vegetation structural complexity and vertebrate fauna (Bancroft et al., 2008; Delhey, 2018; Lowney & Thomson, 2021; Mainwaring et al., 2015; Natusch et al., 2016). Yet a recent review revealed that very few studies have investigated birds as terrestrial ecosystem engineers, instead a considerable bias towards invertebrates and mammals was observed (Coggan et al., 2018).

Animals living in arid habitats regularly face harsh conditions. Maximum and minimum temperatures can exceed the upper and lower thresholds of many species and precipitation is unpredictable, resulting in fluctuations between scarce or plentiful vegetation cover (Hillel & Tadmor, 1962; Rosenzweig, 1968), which determines the availability of resources to other species further up the food web (Polis, 1991). The impact of any engineer may change depending on these environmental contexts. Using a spatial aridity gradient allows for comparison of species interactions with ecosystem engineers as environmental stress increases. This approach may enhance predictions of how animal community structure and species interactions may change as benign sites become harsher and how engineers could mitigate stress.

**FIGURE 1:**
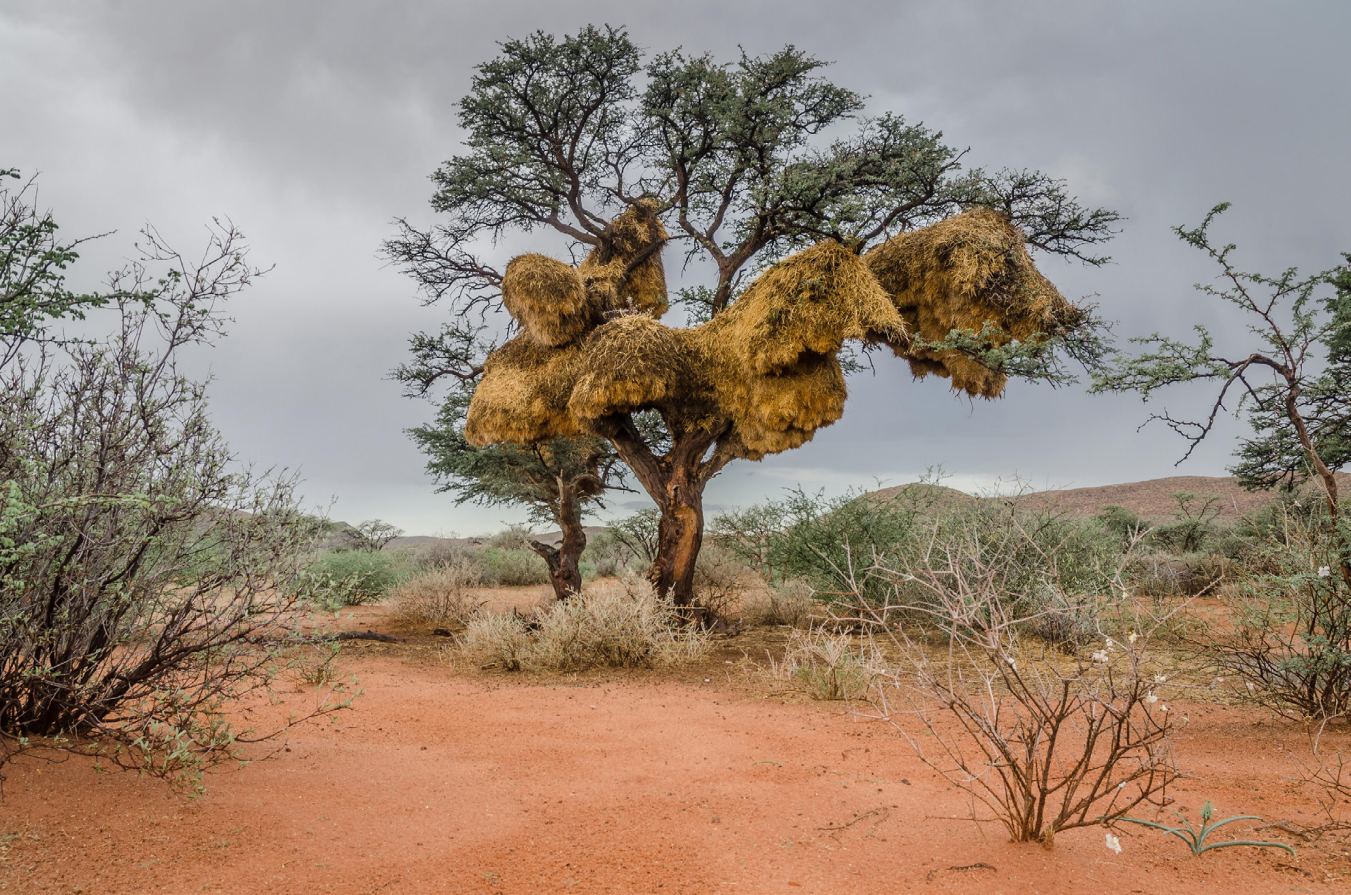
A sociable weaver colony at one of the study sites. This colony was at Tswalu Kalahari Reserve and contained 167 chambers

Our aim is to determine the role of an avian ecosystem engineer on animal species diversity, and we use this system to test how its impacts may change across spatial gradients of environmental harshness. Our focal species is the sociable weaver *Philetairus socius* (henceforth weaver). These small passerines construct large nest colonies and are endemic to the semi‐arid and arid areas of the western parts of southern Africa (Maclean, 1973; Mendelsohn & Anderson, 1997). Colonies can contain hundreds of nesting chambers and are inhabited and maintained year‐round, meaning that some colonies can remain active in the environment for over a century (Maclean, 1973). In addition, colonies are also dynamic and can increase in size from year to year or completely collapse. Larger colonies may host hundreds of individual birds and nest chambers provide insulation, a crucial resource in arid environments, for its occupants (Lowney et al., 2020). Soils directly below colonies have particularly increased nutrient levels (Prayag et al., 2020) and this could result in direct effects on the local vegetation and animals. Weaver colonies have been shown to act as a resource to multiple species within the environment (Bolopo et al., 2019; Lowney & Charlton, 2017; Maclean, 1970; Rehn, 1965; Rymer et al., 2014) and maintain this impact throughout the year (Lowney & Thomson, 2021). Their facilitative role across their distribution remains unknown (Figure 1).

We undertook a meta‐replicated study investigating weaver colony use by local animal communities at sites across a >1,000 km gradient of the weaver's distribution. The eight sites selected differed in aridity and represent a ‘harshness gradient’ that allowed us to test the predictions of the SGH. We hypothesized that because weaver colonies ameliorate harsh abiotic conditions and provide biotic resources, that as conditions became harsher these mechanisms would increasingly buffer animal communities. We expected to observe greater animal diversity of multiple taxa at trees containing a weaver colony compared with control trees without a colony across all sites. Most species that use weaver colonies are not obligate associates and can exhibit behavioural plasticity in their use of weaver colonies. We hypothesize that because invertebrate abundance is influenced by organic matter (Noy‐Meir, 1985) and this collects below colonies (Prayag et al., 2020), that a greater abundance of invertebrates will be observed at colony trees than the control trees. We also hypothesize that due to thermal insulation against hot and cold temperatures that colonies provide (Lowney et al., 2020) and increased resources in terms of invertebrate abundance, small‐ to medium‐sized birds and reptiles will associate with colony trees. We hypothesize that use of colonies for shade and territory marking (Lowney & Thomson, 2021) will increase the associations of large mammals, while herbivores are likely to forage more at colony trees due to the nutrient‐rich vegetation and increased foliar biomass (Prayag et al., 2020). Therefore, we expect increased number of large vertebrates at colony trees. The SGH predicts that positive interactions should be more frequent in communities under high physical stress (Bertness & Callaway, 1994). Therefore, we hypothesize that the relative number of animals that interact with colony trees would increase at sites with lower rainfall and normalized difference vegetation index (NDVI) values, and we predicted that the use of colonies would increase at sites with lower rainfall and lower plant productivity.

## 2 MATERIALS AND METHODS

### 2.1 Study sites

**FIGURE 2:**
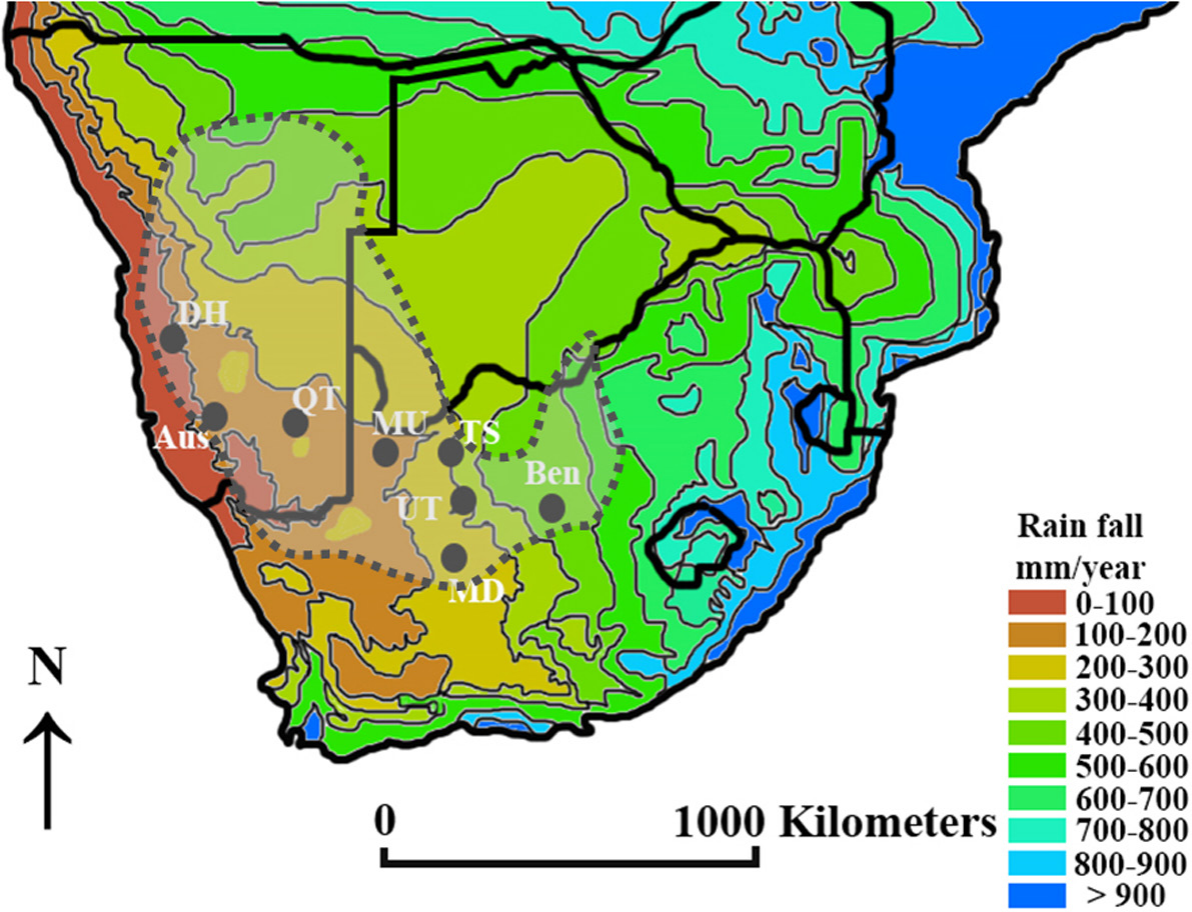
The eight study sites used were selected across a range of annual rainfall. These sites were Desert homestead (DH), Aus (Aus), Quiver tree forest (QT), Murray ranch (MU), Tswalu Kalahari (TS), Uitkoms guest farm (UT), Marydale (MD) and Benfontein (Ben). Dotted lines represent the weaver distribution

We visited eight sites one after the other between March and May 2018 (Figure 2), with five sites in South Africa and three in Namibia. We selected sites to incorporate a rainfall gradient, and we visited these in the following order: Dedeben (‘Tswalu Kalahari’ −27.287, 22.484, mean rainfall per year 361 mm, study area approx. 32 km^2^, colony density 2.34 km^2^), Kimberly (‘Benfontein Nature Reserve’ −28.824, 24.821, 430 mm, 20 km^2^, colony density 0.85 km^2^), Marydale (−29.352, 22.264, 132 mm, 6.5 km^2^, colony density 5.85 km^2^), Askham (‘Murray Ranch’ −26.985, 20.865, 93 mm, 15 km^2^, colony density 1.73 km^2^), Rietkloof (‘Uitkoms guest farm’ −28.544, 22.461, 241 mm, 27 km^2^, colony density 0.48 km^2^), Keetmanshoop (‘Quiver tree forest’ −26.481, 18.243, 138 mm, 15 km^2^, colony density 2.13 km^2^), Aus (−26.561, 16.484, 84 mm, 29 km^2^, colony density 0.69 km^2^) and Sesriem (‘Desert homestead’ −24.661, 15.941, 62 mm, 15 km^2^, colony density 1.80 km^2^; Figure 2). Study sites were a mean distance of 161 km apart (±53.9 *SD*, range 91–237 km).

### 2.2 Survey methods

We carried out surveys to quantify weaver colony association by invertebrates, reptiles, birds and large vertebrates at each site over a 7‐day period. We mapped all sociable weaver colonies within the first 24 hr on each site. From mapped colonies, we selected those found in camel thorn trees, as these trees are frequently used by weavers throughout their distribution. We paired each selected colony tree with a camel thorn tree similar in structure that did not contain a weaver colony (‘non‐colony tree’). To account for spatial effects within sites, paired trees were less than 200 m apart (mean 90 m + 51.5 *SD*). Initially, 16 colony trees and 16 non‐colony trees were selected at each site to reduce disturbance at each tree, with eight pairs used for large vertebrate sampling and eight used to sample other taxa; however, after the first site it became clear that some sites would not contain enough colonies to follow this protocol. Therefore, we performed all sampling at the same eight colony trees and their eight paired non‐colony trees per site.

We recorded the main characteristics of each tree. We measured tree height taking photographs with a reference ladder placed at the tree and using ImageJ software (Schindelin et al., 2012). Trunk diameter was measured as the DBH with a standard tape measure; if the trunk split before this point, the diameter was measured immediately below this split. We calculated canopy cover, by measuring the maximum length and perpendicular width of the canopy and applying these to the equation: canopy cover = (*πr* ^2^)/2, where *r* = (maximum length + perpendicular width)/2 (Witkowski et al., 1994). We used a principal component analysis to reduce correlated tree variables which loaded heavily on principal component 1 (95.8%; Table S1).

We used different sampling methods to target different taxa. In total, seven sampling methods were used to quantify the abundance and diversity of fauna present at each tree with a colony (‘colony tree’) and each tree without (‘non‐colony tree’).

### 2.3 Abundance of invertebrates

We used complementary techniques to sample invertebrate diversity, pitfall traps for terrestrial invertebrates and pan traps for aerial insects. These were deployed at trees and left undisturbed for three consecutive nights. Pitfall traps consisted of 50‐mL falcon tubes (30‐mm diameter), filled with ~20 ml of a 1:1 solution of water and propylene glycol and buried so that the lip of the tube was flush with the soil surface. Six pitfall traps were placed under each tree at 2‐m intervals in a transect running outward and starting at the base of the trunk and directly under the colony. Pan trapping consisted of yellow plastic trays (40 cm length, 30.5 cm width and 8 cm deep) that were half filled with a 1:1 solution of water and propylene glycol. Colour preference of invertebrates can bias sampling, and when undertaking comparative biodiversity studies high reflectance colour trays (white or yellow) should be used (Vrdoljak & Samways, 2012). A single trap was placed on the ground under each tree. At colony trees, traps were placed as near to the colony as possible, without being directly underneath as these traps would quickly fill with faecal matter. When setting traps at colony trees, positions relative to the tree were recorded and used to place traps in same locations at paired non‐colony trees. We stored insects in 99% ethanol until later identification. All individuals were initially identified to morphospecies then to family level and where possible to genus or species. Morphospecies are commonly used in ecological studies and can be used as surrogates for formal species (Vrdoljak & Samways, 2012).

### 2.4 Abundance of reptiles

We followed the protocol by Rymer et al. (2014) to sample reptile abundance. We carried out counts from four locations around a colony tree, each lasting 4 min, using spotting scopes (Kowa TSN‐881 and Kowa 20‐60x eyepiece). The observer was located at least 50 m from the tree, and after each 4‐min count, the observer moved to the next location, approximately 90 degrees around the tree. Once a full rotation of the tree had been completed, a further 4 min were used for the observer to walk towards and around the tree slowly; this helped identify individuals on the ground, and to clarify any uncertainties about whether some individuals may have been counted twice. Counts were carried out simultaneously by two different observers, at the colony tree and at the paired non‐colony tree. The observers rotated between counting colony and non‐colony trees. Only two species of reptiles were observed during these counts: the Kalahari tree skink *Trachylepis spilogaster* and the variegated skink *Trachylepis variegata*. The distribution ranges of these species only overlapped at three of the eight sites; therefore, we only compared reptile abundance and not species richness or diversity.

### 2.5 Abundance of avian species

We estimated avian diversity using a single 4‐min point count at colony and non‐colony trees. We recorded all birds seen or heard within 25 m of the tree. Colony trees were paired with different non‐colony trees to those mentioned above, here being a minimum of 200 m and a maximum of 500 m away from colony trees (range 201–474; mean + 57.49 *SD*). This was to reduce the disturbance of an observer and to reduce the likelihood of the same bird being counted at colony and non‐colony trees. Trees were matched for tree species and size. A single observer conducted the point counts at paired trees directly after each other, and the order was rotated between different pairs. Point counts started at sunrise, and the final point count on a given day started within 3 hr of the first count.

### 2.6 Abundance of roosting birds

We followed Lowney and Thomson's (2021) protocol to sample roosting bird abundance and carried out nocturnal surveys at weaver colonies to document heterospecific birds roosting in weaver chambers. All colonies at a given site were visited on the same night, with surveys starting at least 30 min after sunset. We used a head torch to scan chamber entrances, spending no more than 5 min at a given colony; birds that roosted in chambers were seen and identified. We used data from the point counts mentioned above to account for the local bird community when analysing numbers/species of bird roosting in weaver colonies.

### 2.7 Abundance of vertebrates

We followed the protocol by Lowney and Thomson (2021) to survey vertebrates at colony and non‐colony trees. Camera traps, equipped with an 8 GB memory card and black flashlight, were placed at paired trees concurrently for 5 consecutive days. Camera traps were triggered by motion and programmed to take three consecutive photographs and a 10‐s video. One minute would then elapse before the camera could be triggered again. Cameras at paired trees were always of the same make and model with identical sensitivity settings.

We deployed two cameras at each tree. The first was fitted to a metal stake set 60 cm above the ground and positioned so that it had an unobstructed view of the area below the tree and the nearby vegetation. The second was attached to the tree so that it had an unobstructed view along the top of the weaver colony. If this was not possible, cameras were attached to the trunk and positioned so that they had a clear view along the branch towards the colony. Weavers usually build their colonies on one of the trees' thickest branches (Lowney & Thomson, 2021); therefore, at non‐colony trees we fitted camera traps to the trunk so that they had an unobstructed view along the thickest branch of the tree, this being the branch thought most likely to hold a weaver colony if weavers were to build in the non‐colony tree (Lowney & Thomson, 2021).

The images and videos captured were used to extract the number of events at a given tree. An event was defined as the moment an animal came into view until it left (Lowney & Thomson, 2021). When multiple conspecifics came into view, the event was defined from the moment the first conspecific entered the frame until the last one left. If multiple species were recorded, then separate events were assigned to each species. During each event, we calculated the event duration to the nearest minute and recorded the species and the number of individuals. We removed all events where individuals that were deemed to ‘pass by’ and not interact with the tree and/or colony from the analyses. If individuals triggered both terrestrial and arboreal cameras, we only then used the data from the arboreal camera (Lowney & Thomson, 2021).

### 2.8 Colony use across a spatial gradient

We used rainfall and NDVI as measures of aridity and environmental stress at each site. Water availability is key to environmental productivity and is often used as an indicator of environmental stress (Barchuk & Díaz, 2005; Coggan et al., 2016). Up to 40% of the earth's surface is classified as arid (Salem, 1989), and rainfall can vary considerably within dry environments (Sharon, 1972). Plants in arid environments respond strongly to rainfall events, but these are often unpredictable, meaning that plant productivity can also be variable (Hillel & Tadmor, 1962; Rosenzweig, 1968). NDVI can be used to calculate terrestrial vegetation conditions (Solano et al., 2010) and provide a practical estimate of net primary productivity (Goward et al., 1994). Therefore, NDVI associates positively with local food availability (Hurlbert & Haskell, 2003) and can be used as a proxy for harshness (Goward et al., 1994). NDVI is calculated from reflectance in the near‐infrared and red portions of the electromagnetic spectrum (Hurlbert & Haskell, 2003), and is a measure of greenness that allows for analyses of terrestrial vegetation conditions (Solano et al., 2010). We calculated NDVI values using MODIS time‐series images taken by the satellite Terra (MOD09A1 V6). Each image had a resolution of 500 m and was downloaded for each site for the dates we visited. We calculated NDVI values for each set of paired trees using the GPS location of the colony tree in ArcGIS 10.4 (Lowney & Thomson, 2021).

NDVI values will be higher at a site if it has received recent rainfall. Therefore, for understanding the long‐term harshness of each site we also used the total rainfall for the 24 months before our visit. Rainfall for South African, Keetmanshoop, Aus and Sesriem's Desert homestead sites was provided by the South African Weather Services, Namibian Meteorological Service, Klein‐Aus Vista and NamibRand Nature Reserve respectively. We used a Pearson's correlation to test for collinearity between average temperature and NDVI. The correlation coefficient was >0.7 (*r* = 0.81).

### 2.9 Statistical analyses

We used the R statistical package 3.6.3 to analyse the data (R Core Team, 2017). Following our statistical approach in Lowney and Thomson (2021), we used the glmmTMB package to perform GLMMs and linear mixed models (LMMs) as it can handle zero‐inflated models (Brooks et al., 2019). For overdispersed data, we fitted models with a quasi‐Poisson or negative binomial distribution, and when these still could not control for overdispersion, we transformed data and used a Gaussian distribution (Ives, 2015). We applied zero inflation when needed. We checked the model assumptions using the dispersal estimate parameter in glmmTMB, selecting the distribution with parameter estimates nearest to 1.0. All model details are listed in Table S2. Our model selection used Akaike criterion corrected for small sample size (AICc) with maximum likelihood estimation to compare a sequence of models to determine which best explained the patterns of variation in our data (Harrison et al., 2018). We used the MuMin package to average the models that came within two AICc units of the top model (Barton, 2020). We considered model terms with confidence intervals not intersecting zero to explain significant variation in the data.

Unless otherwise stated, we used ‘tree’ as the sampling unit (each response variable was calculated per tree), to evaluate our response variables (see below). We used colony presence (present/absent, our primary variable of interest), the stress components (NDVI or rainfall) and the tree characteristics (PCA1 loading score) as explanatory variables in each model (Lowney & Thomson, 2021). Due to the strong correlation between NDVI and rainfall, both stress components could not be used in the same model. Therefore, we ran separate models for each response variable—one containing NDVI and one containing rainfall. We fitted an interaction between colony presences and the environmental ‘stress’ components to understand if animals were influenced differently by colony and non‐colony trees depending on the environmental stress (Lowney & Thomson, 2021). Each paired tree was assigned a unique pair ID and each site was given a unique site ID and used as random effects, with pair ID nested with site ID in each model. We also scaled the stress component and tree characteristic variables. We included all explanatory variables and random effects in each model unless otherwise stated.

#### 2.9.1 Abundance of invertebrates

For both terrestrial and aerial invertebrates we used the number of individuals, species richness and Shannon–Weiner diversity index (hereafter, Shannon diversity) as our response variables, calculated using the vegan package (Oksanen et al., 2017). For terrestrial invertebrates, a negative binomial GLMM was used to investigate the number of individuals, whereas data transformation was applied to species richness (log) and Shannon diversity (square root), and these were analysed using an LMM. For aerial invertebrates, a quasi‐Poisson‐fitted GLMM was used to investigate the number of individuals and a Gaussian LMM was used to investigate Shannon diversity. Data transformation (log) was used for species richness and analysed using an LMM.

#### 2.9.2 Abundance of reptiles

We used the total number of reptiles observed as our response variable. This GLMM was fitted with a Poisson distribution.

#### 2.9.3 Abundance of avian species

The total number of individuals, species richness and Shannon diversity were used as response variables. GLMMs with negative binomial and Poisson distributions were used to compare the total number of individuals and species richness respectively. Data transformation was applied to Shannon diversity (square root) and analysed using an LMM. Sociable weaver observations were removed all from the point count data (Lowney & Thomson, 2021).

#### 2.9.4 Abundance of roosting birds

To investigate heterospecific roosting in colonies, we used the following response variables: colonies occupied by at least one heterospecific species (yes/no), the number of non‐sociable weaver species, species richness and Shannon diversity. Our stress variables and colony size (number of chambers) were used as explanatory variables. The number of conspecifics that were recorded during the point counts were used as an offset variable in the model. Site ID was used as a random term. Due to there being no interactions in these models, we opted to test full models without the implementation of AIC and model selection. GLMMs investigating the probability that a colony is occupied were fitted with a binomial distribution, whereas those investigating the number of heterospecific species and species richness were fitted with negative binomial and Poisson distributions respectively. Data transformation was applied to Shannon diversity (square root), and these were analysed using an LMM (Lowney & Thomson, 2021).

#### 2.9.5 Abundance of vertebrates

The response variables we used were the number of camera trap events, event duration, species richness and Shannon diversity. To analyse the event duration, we used each ‘event’ captured as the sampling unit. We used negative binomial‐distributed GLMMs to compare the number of camera trap events and event duration, quasi‐Poisson to compare species richness and Gaussian to compare Shannon diversity (Lowney & Thomson, 2021).

### 2.10 Ethics

The project conformed to the legal requirements of South Africa and Namibia and received research permits from the South African Northern Cape Province's Department of Tourism and Environment and Conservation (ODB 2516/2016 and ODB 0007/2017), Namibia's National Commission on Research, Science and Technology (Permit Number RPIV00082018) and an ethics approval from the University of Cape Town, South Africa (2015/V14/RT).

## 3 RESULTS

Of the 21 model sets that used rainfall as the stress gradient component and the 21 that used NDVI, all but six produced similar results. Therefore, unless results differ by at least one level of significance (*p* < 0.05, *p* < 0.01 or *p* < 0.001), then we only present the results from the rainfall model sets. However, the results of all models can be found in the supplementary materials. Below we only present significant results, all other results can be found in Tables S3–S24. Again, we only present figures where statistical differences were observed, and here we use predicted values to demonstrate the effect sizes caused by those response variables that produced significant differences. Therefore, we did not produce figures for each taxon. However, we see this study and these statistical tests as replicated tests of the SGH and that figures for each taxon are not required, despite the taxa being obviously interconnected.

### 3.1 Abundance of terrestrial invertebrates

In total, we placed 384 pitfall traps each at colony and non‐colony trees. We used the 319 trap pairs that were left undisturbed for analyses. In all, 141 morphospecies (mean ± *SE* 3.01 per trap ±0.08) and 5966 (mean per trap 9.32 ± 0.46) invertebrates were captured in pitfall traps.

**FIGURE 3:**
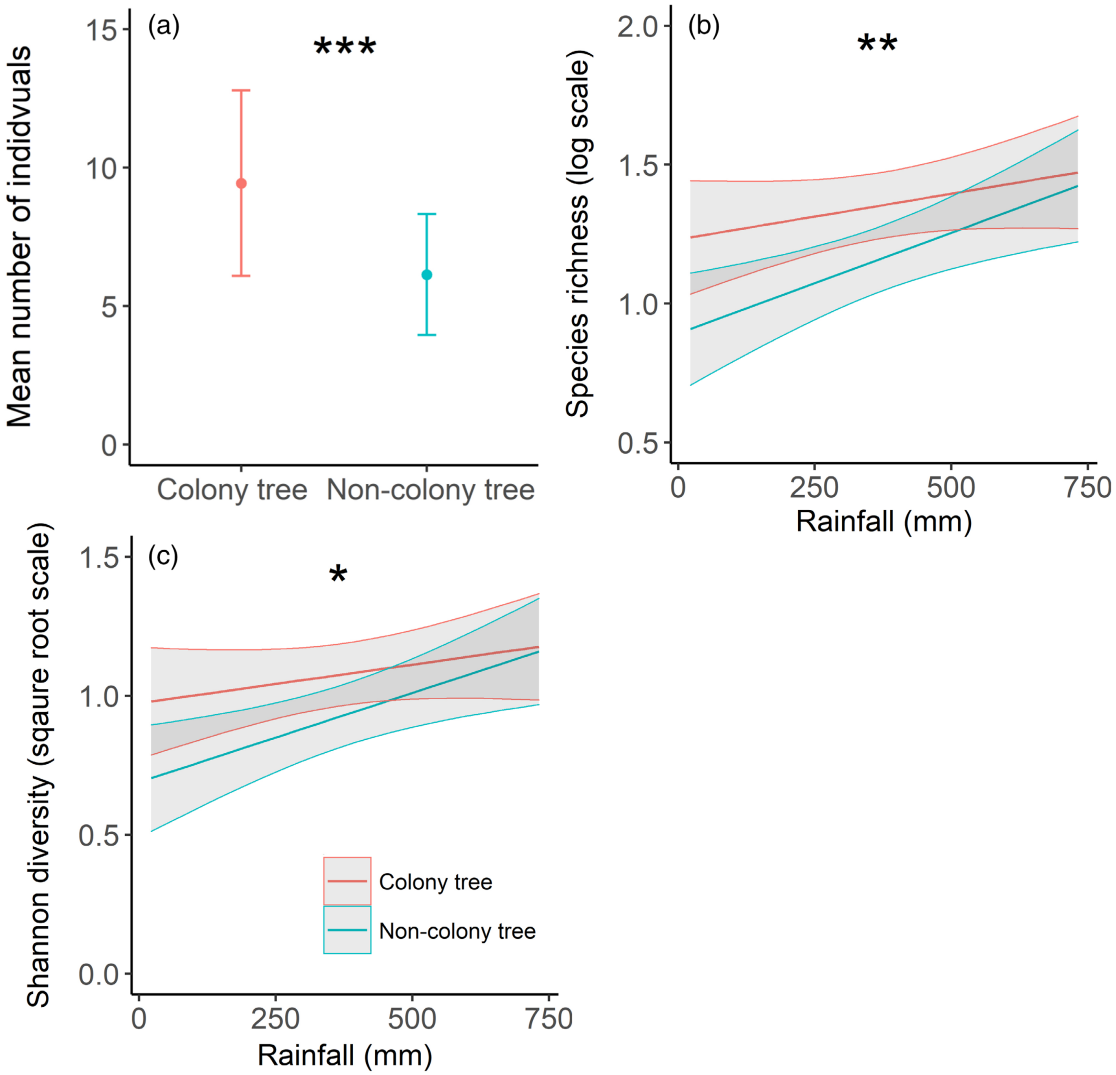
Model predictions (±95% confidence limits) from the top‐ranked competing models for (a) the number of terrestrial invertebrates captured in pitfall traps at colony and non‐colony trees, (b) for species richness at colony and non‐colony trees relative to rainfall and (c) Shannon diversity at colony and non‐colony trees relative to rainfall (*p < 0.05, **<0.01, ***<0.001)

Five competing models explained the number of individuals in pitfall traps in our rainfall model dataset (Table S3). Model‐averaged coefficients revealed that colony presence was the only variable whose 95% confidence limits (CLs) did not overlap zero (*z* = 5.588, *p* < 0.001, Table S4), with 54% more invertebrates at colony trees (Figure 3a). Two and five competing models explained species richness and Shannon diversity respectively (Table S3). For both response variables, the interaction between colony presence and rainfall had CLs that differed from zero (Table S4). The models revealed a positive relationship with rainfall at non‐colony trees, whereas no relationship was observed at colony trees (Table S4; Figure 3b,c).

**FIGURE 4:**
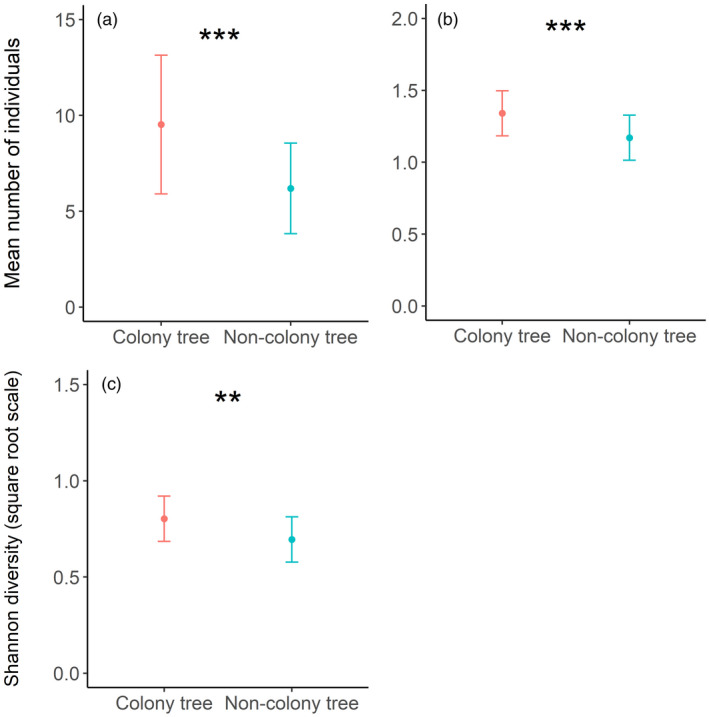
Model predictions (±95% confidence limits) from the top‐ranked competing models for the relationship between terrestrial invertebrates at colony and non‐colony trees from models that used NDVI as the environmental stress component for: (a) the number of individuals, (b) species richness and (c) Shannon diversity. Predictions (**p < 0.01, ***<0.001)

For model sets containing NDVI as the stress gradient, two competing models explained the number of invertebrate individuals and five explained species richness and Shannon diversity (Table S5). For all models, averaged coefficients revealed that colony presence was the only variable whose CLs did not overlap zero: number of individuals (*z* = 5.578, *p* < 0.001), species richness (*z* = 5.277, *p* < 0.001) and Shannon diversity (*z* = 3.258, *p* = 0.001, Table S6). At colony trees, the number of individuals increased by 54% (Figure 4a), species richness increased by 15% (Figure 4b) and Shannon diversity increased by 16% (Figure 4c).

### 3.2 Abundance of aerial invertebrates

In total, we placed 64 pan traps each at colony and non‐colony trees; of these 59 pairs were left undisturbed and used for analyses. We captured 132 morphospecies (mean 7.16 per trap ±0.31) and 2704 (mean per trap 17.58 ± 12.8) invertebrates.

**FIGURE 5:**
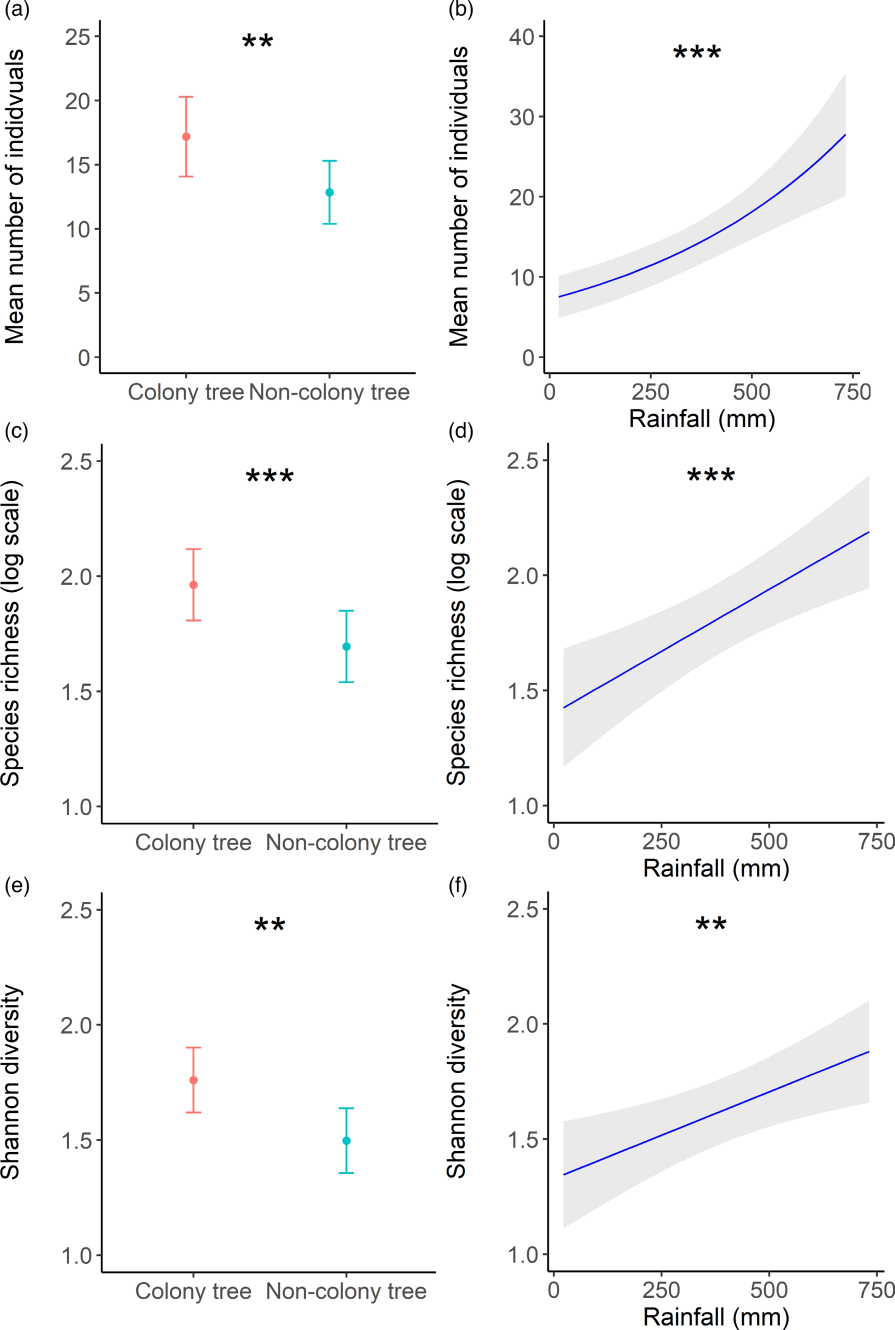
Model predictions from the top‐ranked competing models (±95% CI) for (a, b) the mean number of individuals, (c, d) species richness and, (e, f) Shannon diversity for invertebrates collected in pan traps at (a, c, e) colony and non‐colony trees and (b, d, f) the previous 24 months' rainfall. (**p < 0.01, ***<0.001)

Four competing models explained the number of individuals, while two competing models explained species richness and Shannon diversity (within ∆AICc = 2.0; Table S7). Model‐averaged coefficients revealed that colony presence and rainfall were the only variables where CLs did not overlap zero (Table S8). Colony trees had 33% more individuals (*z* = 3.274, *p* = 0.001, Figure 5a), 15% greater species richness (*z* = 3.702, *p* < 0.001, Figure 5c) and 18% greater Shannon diversity than non‐colony trees (*z* = 3.897, *p* = 0.001, Figure 5e, Table S8). Increasing rainfall across the range (22–732 mm) was associated with nearly four times as many individuals (*z* = 5.218, *p* < 0.001, Figure 5b), 54% greater species richness (*z* = 3.724, *p* < 0.001, Figure 5d) and 40% greater Shannon diversity (*z* = 2.910, *p* = 0.004, Figure 5f; Table S8). For NDVI model sets, NDVI did not affect Shannon diversity (Tables S9 and S10).

### 3.3 Abundance of reptiles

Three competing models explained the number the of reptiles observed at a given tree in each of the NDVI and rainfall model sets (Tables S11 and S13). Model‐averaged coefficients revealed that colony presence from the NDVI model set was the only variable where CLs did not overlap zero (*z* = 0.213, *p* = 0.027, Table S12), with nearly twice as many reptiles on colony trees. Confidence limits for colony presence in the rainfall model set overlapped zero by 0.013 (*z* = 1.901, *p* = 0.056, Table S14).

### 3.4 Abundance of avian species

We conducted 64 point counts at both colony and non‐colony trees. In all, 34 unique species (mean 0.7 species/count ±0.08) and 119 individuals (mean 0.93 individuals/count ±0.12) were observed.

**FIGURE 6:**
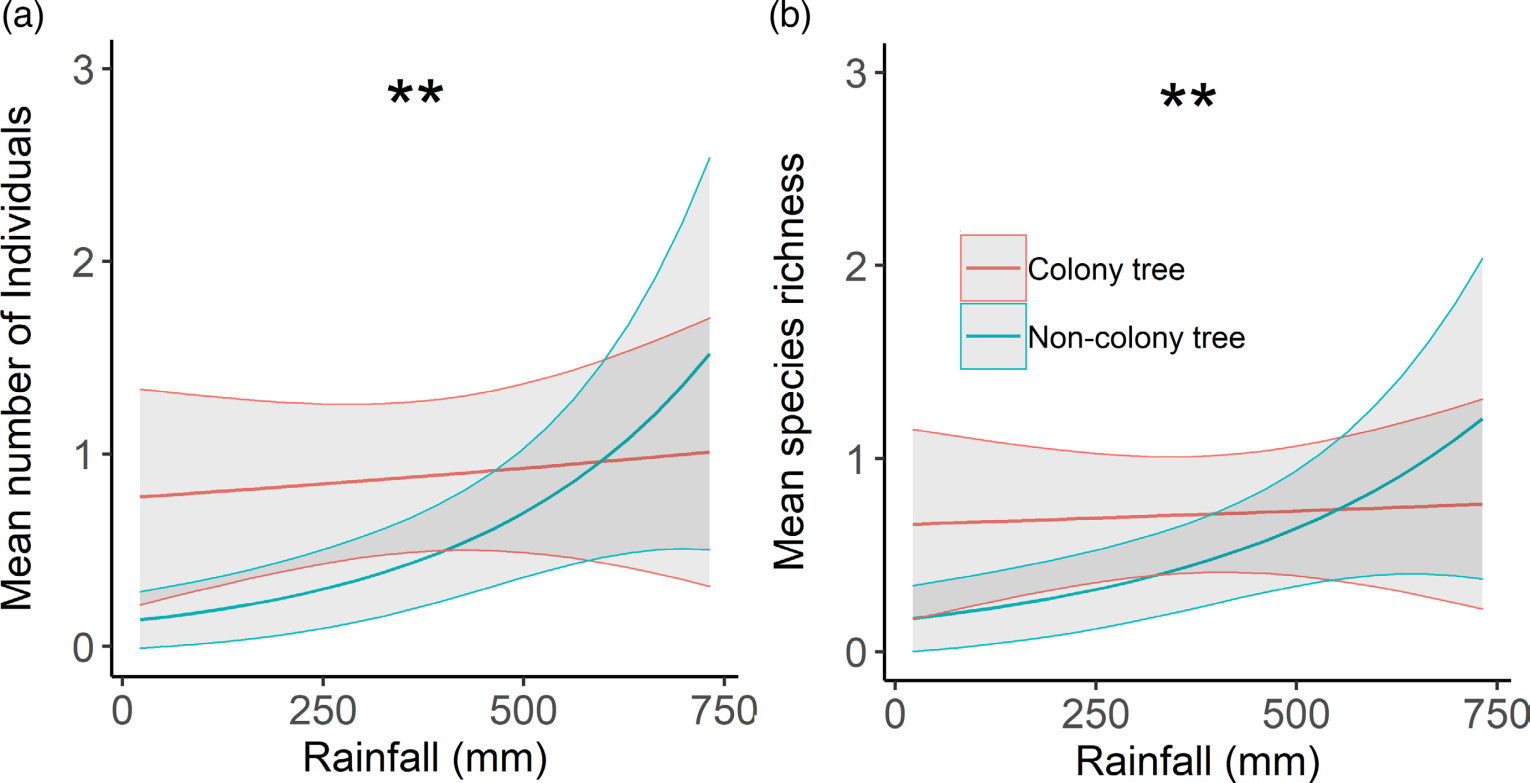
Model predictions (±95% CI) from the top‐ranked competing models of the relationship between colony and non‐colony trees relative to rainfall: (a) for the number of birds and (b) bird species richness. (p** < 0.01)

Two competing models explained the number of individual birds and one explained species richness (Table S15). Model‐averaged coefficients revealed that CLs of the interaction between colony presence and rainfall differed from zero, for number of individuals and species richness (Table S16), and that a positive relationship with increasing rainfall at non‐colony trees, but not at colony trees, was observed for the number of individuals (*z* = 3.101, *p* = 0.002, Table S16; Figure 6a) and species richness (*z* = 2.660, *p* = 0.006, Table S16; Figure 6b). Four competing models explained Shannon diversity; however, none of the explanatory variables explained significant variation (Tables S15 and S16).

For models using NDVI as the environmental stress variable, three competing models explained the number of individuals, species richness and Shannon diversity (Table S17). None of the explanatory variables explained significant variation in any of the response variables (Table S18).

### 3.5 Abundance of roosting birds

In total, 33 of the 64 (51%) nest colonies hosted heterospecific roosting birds. Six species were recorded: African pygmy falcon, Acacia pied barbet *Tricholaema leucomelas*, ashy tit *Melaniparus cinerascens*, scaly‐feathered finch *Sporopipes squamifrons*, red‐headed finch *Amadina erythrocephala* and Cape sparrow *Passer melanurus*. A mean of 1.53 ± 0.25 heterospecific individuals were observed per colony.

**FIGURE 7:**
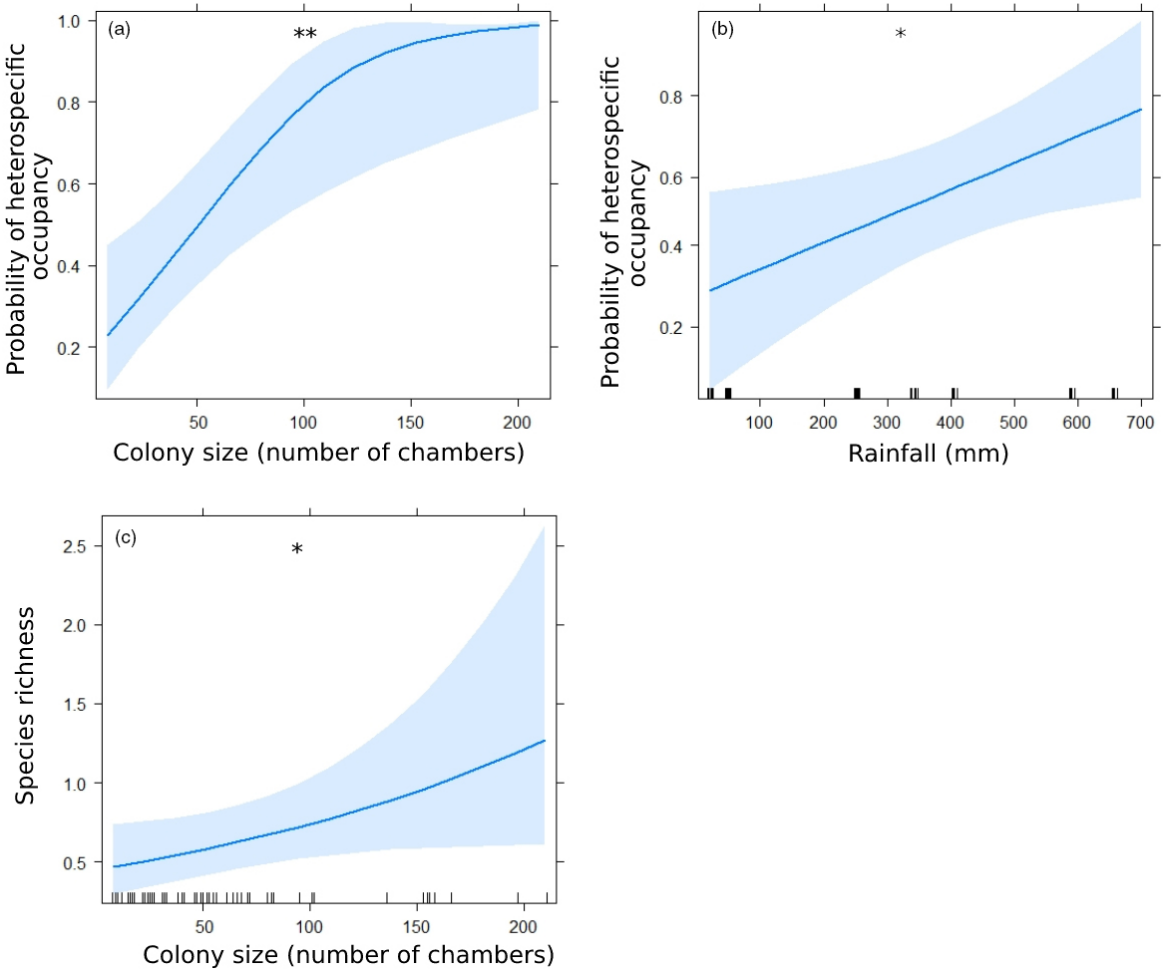
Model predictions for roosting heterospecifics (±95% CI; *p < 0.05, **<0.01): (a) the likelihood of colonies being occupied by heterospecifics, in relation to colony size (b) and rainfall, and (c) the relationship between the species richness and colony size

Colony size and rainfall increased the probability that colonies hosted roosting heterospecifics. Larger colonies were four times more likely to contain heterospecifics (colony size range: 8–211; *χ* ^2^ = 8.008, *p* = 0.005, Table S19; Figure 7a), and the probability more than doubled at colonies in areas with higher rainfall (rainfall range: 22–732; *χ* ^2^ = 5.336, *p* = 0.021, Table S19; Figure 7b). The size of a colony also increased species richness, with nearly three times as many species recorded in larger colonies (*χ* ^2^ = 3.89, *p* = 0.049, Table S19; Figure 7c).

### 3.6 Abundance of vertebrates

We observed 49 unique species over a total of 1,280 camera trap days. On average, we recorded 2.02 ± 0.17 species per tree for the 5‐day sampling period. A total of 314 events were captured (mean events per tree = 2.45 ± 0.21), and event durations averaged 34.38 min ± 8.68 per tree.

**FIGURE 8:**
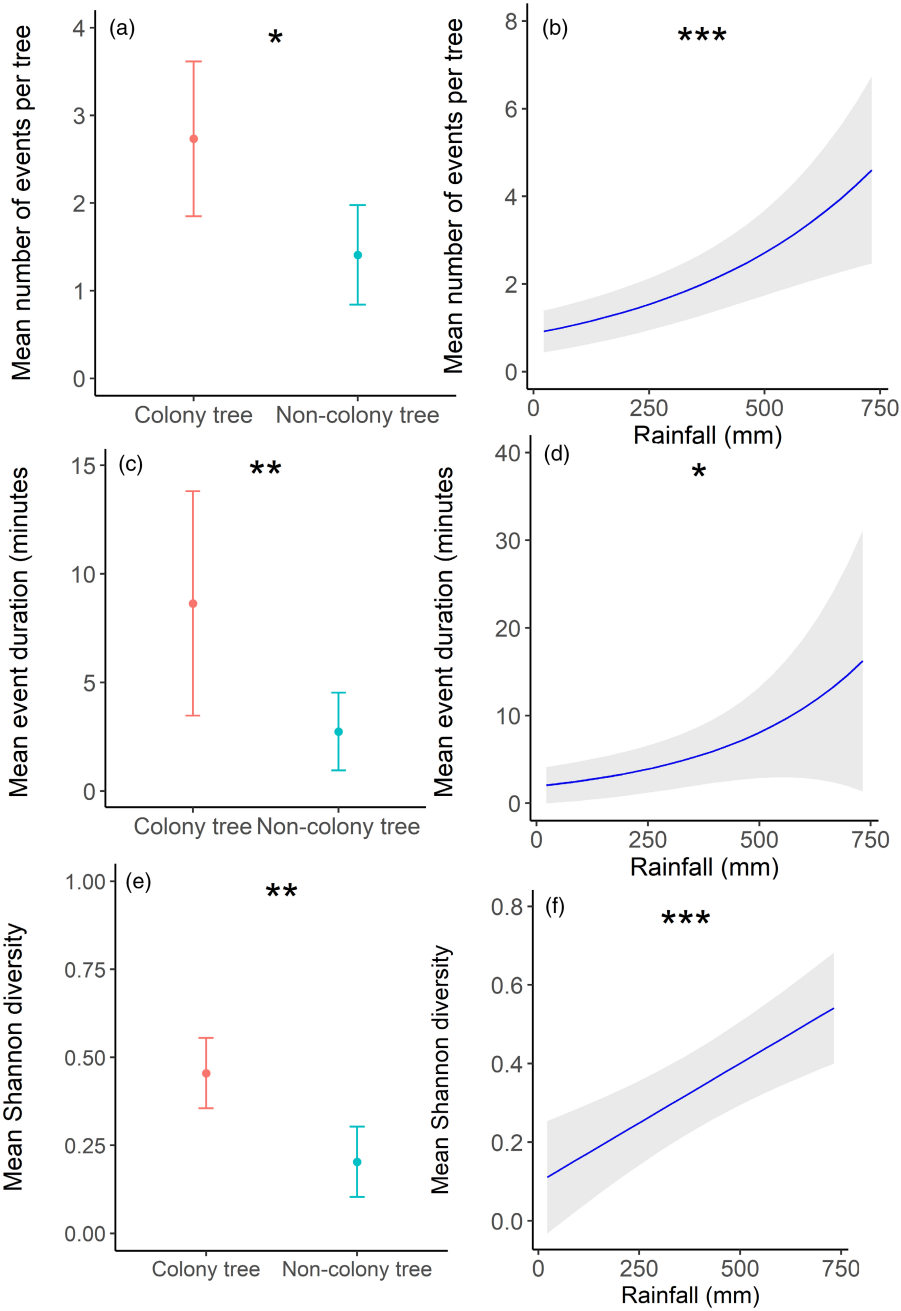
Model predictions (±95% CI) from the top‐ranked competing models for (a, b) the mean number of events, (c, d) event duration and (e, f) Shannon diversity, captured by camera traps of vertebrates between: (a, c, e) colony and non‐colony trees and (b, d, f) the previous 24 months' rainfall. Predictions (*p < 0.05, **<0.01, ***<0.001)

Three competing models explained the number of camera trap events and species richness, while two competing models explained event duration and Shannon diversity (Table S21). For all models, except species richness, averaged coefficients revealed that colony presence and rainfall were the only variables where CLs did not overlap zero (Table S22). In species richness models, all variables overlapped zero. At colony trees, there were nearly twice as many events (*z* = 3.010, *p* = 0.027, Table S22; Figure 8a), events lasted more than three times longer (*z* = 3.088, *p* = 0.002, Table S22; Figure 8c) and Shannon diversity more than doubled compared to non‐colony trees (*z* = 3.437, *p* = 0.001, Table S22; Figure 8e). Increasing rainfall was associated with five times more animal events (*z* = 4.525, *p* < 0.001, Table S22; Figure 8b), eight times longer event durations (*z* = 2.370, *p* = 0.018, Table S22; Figure 8d) and five times greater Shannon diversity (*z* = 4.148, *p* < 0.001, Table S22; Figure 8f), across the rainfall range (rainfall range = 22–732 mm).

For models using NDVI as the environmental stress variable, two competing models explained the number of events, two explained event duration, four explained species richness and four explained Shannon diversity (Table S23). However, results from these models did not differ from those that contained rainfall as the environmental stress variable (Table S24).

## 4 DISCUSSION

Our study demonstrates that weaver colonies enhance local diversity across their range and that these strong associations are a replicated and consistent feature of these structures. For all taxa sampled (invertebrates, birds, reptiles and mammals), we found increased numbers of individuals associated with camel thorn trees hosting weaver colonies. Furthermore, we observed increased species richness in invertebrates and roosting birds, and increased species diversity in invertebrates and vertebrates associated with camel thorn trees hosting weaver colonies. Likely through a variety of mechanisms, weaver colonies create habitat and enhance resources to surrounding animal communities, serving as an ecological engineer. Importantly, our results suggest that weaver colonies were associated with increased relative numbers of animal events and diversity especially at the more arid sites for both birds and terrestrial invertebrates. Camel thorn trees containing weaver colonies maintained higher biodiversity and animal associations despite increasing aridity at sites, in contrast to non‐colony trees where overall animal diversity and associations decreased as aridity increased. This provides support for a prediction of the SGH, where facilitation by these ecosystem engineers becomes increasingly important in animal communities as harshness of the environment increases.

### 4.1 Abundance of invertebrates

Both terrestrial and aerial invertebrates demonstrated a strong association with colony trees, which was maintained at the more arid sites. Weaver colonies clearly provide an environment and resources suitable for invertebrate diversity. Additionally, aerial invertebrates saw an increase in the number of individuals, species abundance and species richness at wetter sites. Factors key to insects in arid environments are temperature, water availability and the presence of organic matter (Noy‐Meir, 1985). Organic matter collects underneath colonies in the form of fallen nest material and with potentially hundreds of avian residents, faeces deposited can form substantial faecal mats that would be an essential source of organic matter in these environments (Prayag et al., 2020). The more arid an environment, the greater the importance of this source of organic matter would be, and this would explain the difference between terrestrial invertebrate interactions with colony and non‐colony trees across the environmental gradient. Invertebrates form an important resource for other taxa too, therefore their strong association may feedback into further positive associations of other taxa with colony trees. Not only does this add further support for the importance of birds as ecosystem engineers, but this also demonstrates that a particular species can become more important to the local animal community as environmental harshness increases, supporting the SGH. Furthermore, this is evidence that the SGH can be tested across animal communities and not confined to single pairwise species interactions.

### 4.2 Abundance of reptiles

Colony presence increased the abundance of reptiles. Our findings support previous research that showed reptiles associate with weaver colonies (Rymer et al., 2014). However, we did not see variation between the differences in colony and non‐colony trees across the aridity gradient. The main factors that influence the abundance of reptiles are food availability and the ability to thermoregulate (Corbalán et al., 2013), and colonies provide shelter and resources. The two tree skink species observed (*Trachylepis* spp.) are insectivores, and invertebrate abundance is higher around colony trees. Therefore, colonies provide the primary resources needed by reptiles in arid environments and explain why colony trees have greater interactions with reptiles than non‐colony trees across all sites. The increase in numbers at wetter sites is likely driven by productivity leading to an increase in prey. Given that only two reptile species were observed, it may be more applicable to install pitfall traps with drift fences around colony and non‐colony trees to determine the response of this taxa more reliably (Larsen, 2016).

### 4.3 Abundance of avian species and roosting birds

Birds showed an association for colony trees in more arid sites. In harsh environments, small‐ to medium‐sized birds avoid high temperatures by seeking refuge (Willmer et al., 2009). Weaver colonies should provide permanent shade and the chambers have been shown to buffer against harsh temperatures (Lowney et al., 2020; Lowney & Thomson, 2021). All the birds observed during point counts were small‐ to medium‐sized birds, suggesting that this kind of facilitation would explain the differences observed between the interactions with colony and non‐colony trees across the environmental gradient. Additionally, insects are an essential nutrition and water source for many bird species. Therefore, strong invertebrate associations found at weaver colonies may explain why more birds associate with colony trees in areas of low rainfall.

Our results also showed the importance of colonies for roosting birds; 51% of colonies hosted heterospecifics on the single sampled night. Larger colonies were more likely to attract heterospecifics and had a greater species richness of roosting birds. More chambers mean more available buffered ‘resources’ for other species to use as roosting sites. In addition, roosting with hundreds of other heterospecifics may reduce the risk of predation via the dilution effect (Beauchamp, 1999). Our quantitative data, together with anecdotal data, show weaver colonies are important to several bird species; rosy‐faced lovebird *Agapornis roseicollis* breed in weaver colonies (Ndithia et al., 2007), while other raptor species have been observed using weaver colonies as platforms to nest on (Oschadleus, 2019).

### 4.4 Abundance of vertebrates

Mammals associated with colony over non‐colony trees, but we did not detect that the relative importance of colony trees increased across the aridity gradient. This lack of impact across the gradient in vertebrates could be explained by how engineers provide facilitation towards different taxa. Many of the terrestrial and arboreal mammals captured by camera traps are species that would need to reduce their metabolic heat production by reducing activity during the hottest parts of the day (Willmer et al., 2009). We have previously found that mammals use weaver colonies for shade (Lowney & Thomson, 2021). However, this study did not include the hottest time of the year and as a result this behaviour was not frequently observed. We speculate that during summer‐time sampling, the increased importance of shade and moisture as a resource would increase the relative importance of colony trees in drier and hotter sites. Furthermore, smaller sample sizes relative to invertebrate and avian data may result in a false negative result. We suggest further studies with a recommendation to increase sample sizes, different seasons and possibly over more extended periods (e.g. camera trapping for a minimum of 2 weeks, Larsen, 2016). Smaller sample sizes also meant that we combined the terrestrial and arboreal data, as a result these were not tested independently.

### 4.5 Colony use across a spatial gradient

The positive diversity impacts of colonies were most pronounced on birds and terrestrial invertebrates at sites with low rainfall. Colony presence increased the number of individuals and species richness at low rainfall sites relative to non‐colony trees; however, the relative difference almost disappeared when environmental conditions became less severe. Thus, the net effect of colonies changed from positive associations of animal communities in the more stressful sites to almost neutral at sites that were benign. This study provides empirical support for the SGH and complements recent research using terrestrial animal communities that found evidence of increased importance of associative or facilitative interactions in harsh environments (Bell & Cuddington, 2019; Dangles et al., 2018; García‐Navas et al., 2021). Additionally, by demonstrating that some taxa associate strongly with colony trees in more arid climates, we provide support that facilitation by weaver colonies increases the realized niches of certain species (Armas et al., 2011; He & Bertness, 2014). Furthermore, only one other study has extended this hypothesis to free‐ranging animals (García‐Navas et al., 2021), as many of the previous studies were tested under laboratory conditions (Bell & Cuddington, 2019; Dangles et al., 2018). Temporal variations in climatic conditions may also play a part in understanding the facilitative role of weaver colonies. This we did not test, but a previous study failed to demonstrate any variation in the use of weaver colonies by heterospecifics across a calendar year (Lowney & Thomson, 2021). However, due to the unpredictability of weather events throughout the weaver's range, this time frame may still be too short to observe variation across a temporal gradient (Lowney & Thomson, 2021). Our site visits were not carried out concurrently, and therefore may have some effects on our results. However, our study design of using paired trees should minimize these impacts. It is likely that such facilitation plays an important role, allowing multiple taxa to persist in environments that may otherwise be too harsh.

Weaver colonies provide different resources for different taxa and, in turn, create ecological hotspots around colony trees. Consequently, colony presence at more stressful sites likely causes more isolated hotspots of life. Many species in harsh habitats have adaptations that allow them to survive with the extreme stresses associated with these environments (Bennett et al., 1984; Schmidt‐Nielsen et al., 1969; Williams & Tieleman, 2005). However, many of the species sampled use colonies as thermal refuges (Lowney & Thomson, 2021). Therefore, our results suggest that, in a landscape that will become increasingly harsh as climate change advances (Akoon et al., 2011), these colonies will become critical ecosystem components that will buffer some of the harsh environmental conditions and allow some species to persist despite these tougher conditions. To conserve biodiversity and reduce the impacts of climate change, it is important to understand how facilitation within animal communities is undertaken and how the processes and interactions within ecosystems are maintained (Coggan et al., 2018). If external temperatures exceed a threshold that colonies can no‐longer sufficiently buffer against, this will have severe consequences for animal communities in these areas. We must understand the role that engineers will play on maintaining ecosystems in an environment changing due to human‐induced climate change and that this should be a priority for future research.

## 5 CONCLUSIONS

Our findings add to the increasing literature that ecosystem engineering is an important biological interaction that can relieve stress in harsh environments (Coggan et al., 2018; Hastings et al., 2007; Romero et al., 2015). We also show the key role of sociable weavers as allogenic engineers in structuring animal communities throughout their range. Furthermore, associations with terrestrial vertebrates and birds to weaver colonies increased in more harsh environments supporting predictions of the SGH, suggesting that colonies may mitigate some of the additional stresses experienced by associated wildlife as climate change advances.

## AUTHORS' CONTRIBUTIONS

A.M.L. and R.L.T. designed the research; A.M.L. performed the research and analysed data; A.M.L. and R.L.T. wrote the paper.

## Supporting information


Supinfo

## Data Availability

Data are deposited in ZivaHub Digital Repository https://doi.org/10.25375/uct.19299062.v1. (Lowney & Thomson, 2022).
