# Supplementary material for: Ecological engineering across a spatial gradient: Sociable weaver colonies facilitate animal associations with increasing environmental harshness
Source: J Anim Ecol. 2022 Mar 29;91(7):1385–99. doi: 10.1111/1365-2656.13688 (PMC9544845; doi:10.1111/1365-2656.13688)
Supplement: Supplementary file 1 — Supinfo [file JANE-91-1385-s001.docx]

**SUPPLEMENTARY INFORMATION**

**Ecological engineering across a spatial gradient: Sociable weaver colonies facilitate animal associations with increasing environmental harshness.**

*Anthony M. Lowney*, Robert L. Thomson*

^1^ FitzPatrick Institute of African Ornithology, DST-NRF Centre of Excellence, University of Cape Town, Private Bag X3, Rondebosch 7701, South Africa.

* Corresponding author, e-mail: anthonym.lowney@gmail.com, telephone number: +27 767663406

**TABLE S1:** Loading scores from the Principal Component Analysis (PCA) that incorporates the three tree characteristics measured from each tree in the dataset: height, canopy cover and circumference at chest height.

| **Principle Component** | **Percent variation** |
| --- | --- |
| Loading score 1 | 95.8% |
| Loading score 2 | 4.2% |
| Loading score 3 | 0.0% |

**TABLE S2.** Complete list of model structures used. Each response variable is listed and the full list of explanatory variables, random effects, error distributions used, transformations employed, zero inflations used and overdispersion parameters are detailed for the specific models. All models were run in the glmmTMB package using the glmmTMB function (Brooks et al., 2019).

| **Response variables** | **Model** | **Distribution** | **Explanatory variables** | **Random effects** | **Transformation** | **Zero inflation** | **Overdispersion parameter** | |  |
| --- | --- | --- | --- | --- | --- | --- | --- | --- | --- |
| **Abundance and behaviour of terrestrial vertebrates** | | |  |  |  |  | Rainfall/NDVI |  |  |
| Camera trap events | GLMM | Negative Binomial | Colony present (yes/no) | Site/ Pair ID |  | No | 1.19/1.35 | |  |
|  |  |  | Tree characteristics (PCA) |  |  |  |  | |  |
|  |  |  | Rainfall/NDVI |  |  |  |  | |  |
|  |  |  | Colony present * NDVI |  |  |  |  | |  |
|  |  |  |  |  |  |  |  | |  |
| Event duration (minutes) | GLMM | Negative Binomial | Colony present (yes/no) | Site/ Pair ID |  | No | 0.428/0.431 | |  |
|  |  |  | Tree characteristics (PCA) |  |  |  |  | |  |
|  |  |  | Rainfall/NDVI |  |  |  |  | |  |
|  |  |  | Colony present * NDVI |  |  |  |  | |  |
|  |  |  |  |  |  |  |  | |  |
| Species richness | GLMM | Quasi-Poisson | Colony present (yes/no) | Site/ Pair ID |  | No | 0.07/0.16 | |  |
|  |  |  | Tree characteristics (PCA) |  |  |  |  | |  |
|  |  |  | Rainfall/NDVI |  |  |  |  |  | |
|  |  |  | Colony present * NDVI |  |  |  |  | |  |
|  |  |  |  |  |  |  |  | |  |
| Shannon Diversity | LMM | Gaussian | Colony present (yes/no) | Site/ Pair ID |  | No | 0.165/0.163 | |  |
|  |  |  | Tree characteristics (PCA) |  |  |  |  | |  |
|  |  |  | Rainfall/NDVI |  |  |  |  | |  |
|  |  |  | Colony present * NDVI |  |  |  |  | |  |
|  |  |  |  |  |  |  |  | |  |
| **Reptile abundance** | | |  |  |  |  |  |  |  |
| Number of Reptiles | GLMM | Quasi-Poisson | Colony present (yes/no) | Site/ Pair ID |  | No |  | |  |
|  |  |  | Tree characteristics (PCA) |  |  |  |  | |  |
|  |  |  | Rainfall/NDVI |  |  |  |  | |  |
|  |  |  | Colony present * NDVI |  |  |  |  | |  |
|  |  |  |  |  |  |  |  | |  |
| **Comparison of bird count data at trees with and without colonies** | | | |  |  |  |  | |  |
| Number of birds | GLMM | Quasi-Poisson | Colony present (yes/no) | Site/ Pair ID |  | No | 0.291/0.48 | |  |
|  |  |  | Tree characteristics (PCA) |  |  |  |  | |  |
|  |  |  | Rainfall/NDVI |  |  |  |  | |  |
|  |  |  | Colony present * NDVI |  |  |  |  | |  |
|  |  |  |  |  |  |  |  | |  |
| Species richness | LMM | Poisson | Colony present (yes/no) | Site/ Pair ID |  | No |  | |  |
|  |  |  | Tree characteristics (PCA) |  |  |  |  | |  |
|  |  |  | Rainfall/NDVI |  |  |  |  | |  |
|  |  |  | Colony present * NDVI |  |  |  |  | |  |
| Species diversity | GLMM | Gaussian | Colony present (yes/no) | Site/ Pair ID |  | No | 0.10/0.10 | |  |
|  |  |  | Tree characteristics (PCA) |  |  |  |  | |  |
|  |  |  | Rainfall/NDVI |  |  |  |  | |  |
|  |  |  | Colony present * NDVI |  |  |  |  | |  |
|  |  |  |  |  |  |  |  | |  |
| **Colony influence on the abundance of roosting birds** | | |  |  |  |  |  | |  |
| Occupancy probability | GLMM | Binomial | Colony size |  |  |  |  | |  |
|  |  |  | Rainfall/NDVI |  |  |  |  | |  |
|  |  |  |  |  |  |  |  | |  |
| Heterospecific abundance | GLMM | Negative binomial | Colony size | Site |  | No | 084/0.85 | |  |
|  |  |  | Rainfall/NDVI |  |  |  |  | |  |
|  |  |  | Point counts (offset) |  |  |  |  | |  |
|  |  |  |  |  |  |  |  | |  |
| Species richness | GLMM | Poisson | Colony size | Site |  | No |  | |  |
|  |  |  | Rainfall/NDVI |  |  |  |  | |  |
|  |  |  | Point counts (offset) |  |  |  |  | |  |
|  |  |  |  |  |  |  |  | |  |
| Shannon Diversity | GLMM | Gaussian | Colony size | Site |  | No | 0.08/0.08 | |  |
|  |  |  | Rainfall/NDVI |  |  |  |  | |  |
|  |  |  | Point counts (offset) |  |  |  |  | |  |
|  |  |  |  |  |  |  |  | |  |
| **Abundance of terrestrial invertebrates** | | | |  |  |  |  | |  |
| Number of invertebrates | GLMM | Negative binomial | Colony present (yes/no) | Site/ Pair ID |  | No | 1.42/1.42 | |  |
|  |  |  | Tree characteristics (PCA) |  |  |  |  | |  |
|  |  |  | Rainfall/NDVI |  |  |  |  | |  |
|  |  |  | Colony present * NDVI |  |  |  |  | |  |
|  |  |  |  |  |  |  |  | |  |
| Species richness | GLMM | Gaussian | Colony present (yes/no) | Site/ Pair ID | Log1 + 1 | Yes | 0.176/0.176 | |  |
|  |  |  | Tree characteristics (PCA) |  |  |  |  | |  |
|  |  |  | Rainfall/NDVI |  |  |  |  | |  |
|  |  |  | Colony present * NDVI |  |  |  |  | |  |
|  |  |  |  |  |  |  |  | |  |
| Shannon diversity | GLMM | Gaussian | Colony present (yes/no) | Site/ Pair ID | sqrt | No | 0.171/0.171 | |  |
|  |  |  | Tree characteristics (PCA) |  |  |  |  | |  |
|  |  |  | Rainfall/NDVI |  |  |  |  | |  |
|  |  |  | Colony present * NDVI |  |  |  |  | |  |
|  |  |  |  |  |  |  |  | |  |
| **Abundance of aerial invertebrates** | | |  |  |  |  |  | |  |
| Number of invertebrates | GLMM |  | Colony present (yes/no) | Site/ Pair ID |  | No | 2.74/2.84 | |  |
|  |  |  | Tree characteristics (PCA) |  |  |  |  | |  |
|  |  |  | Rainfall/NDVI |  |  |  |  | |  |
|  |  |  | Colony present * NDVI |  |  |  |  | |  |
|  |  |  |  |  |  |  |  | |  |
| Species richness | GLMM |  | Colony present (yes/no) | Site/ Pair ID | Log1 | No | 0.148/0.142 | |  |
|  |  |  | Tree characteristics (PCA) |  |  |  |  | |  |
|  |  |  | Rainfall/NDVI |  |  |  |  | |  |
|  |  |  | Colony present * NDVI |  |  |  |  | |  |
|  |  |  |  |  |  |  |  | |  |
| Shannon diversity | GLMM |  | Colony present (yes/no) | Site/ Pair ID |  | No | 0.132/0.126 | |  |
|  |  |  | Tree characteristics (PCA) |  |  |  |  | |  |
|  |  |  | Rainfall/NDVI |  |  |  |  | |  |
|  |  |  | Colony present * NDVI |  |  |  |  | |  |

**TABLE S3**. Top models featuring rainfall as the stress component explaining variation in the number of terrestrial invertebrates: number of individuals (a), species richness (b) and diversity captured at colony and non-colony trees. Global model: Colony present (yes/no) * NDVI + tree characteristics (PCA component one). Random terms: individual tree ID nested within colony ID. In total, 128 trees were monitored (64 colony trees, paired with 64 non-colony trees), each having six pitfall traps placed below. Of which, 320 pairs (n = 640) were without interference.

| Model | df | logLik | AICc | ΔAICc | Model weight |
| --- | --- | --- | --- | --- | --- |
| 1. Number of individuals |  |  |  |  |  |
| Colony present | 5 | -2012.369 | 4034.833 | 0.00 | 0.294 |
| Colony present + rainfall | 6 | -2011.359 | 4034.850 | 0.02 | 0.281 |
| Colony present + tree characteristics | 6 | -2012.157 | 4036.446 | 1.61 | 0.127 |
| Colony present * rainfall | 7 | -2011.137 | 4036.452 | 1.62 | 0.127 |
| Colony present + rainfall + tree characteristics | 7 | -2011.163 | 4036.503 | 1.67 | 0.123 |
|  |  |  |  |  |  |
| 1. Species richness |  |  |  |  |  |
| Colony * rainfall | 8 | -465.952 | 948.133 | 0.00 | 0.529 |
| Colony * rainfall + tree characteristics | 9 | -465.169 | 948.624 | 0.49 | 0.414 |
|  |  |  |  |  |  |
| 1. Species diversity |  |  |  |  |  |
| Colony * rainfall | 7 | -368.756 | 751.688 | 0.00 | 0.507 |
| Colony * rainfall + tree characteristics | 8 | -368.108 | 752.444 | 0.75 | 0.348 |

**TABLE S4.** Factors affecting terrestrial invertebrate abundance for models using rainfall as the stress component. Model estimates are averaged across two competing models for the number of daily individuals (a), two competing models for species richness (b) and two competing models for Shannon diversity (c), within ∆AICc = 2.0.

| Model | # Competing models | Predictor | Estimate | Std. error | Adjusted SE | Z value | *p* value | 2.5% CI | 97.5% CI |
| --- | --- | --- | --- | --- | --- | --- | --- | --- | --- |
| a) Number of individuals | 5 | **Colony present** | **-0.429** | **0.077** | **0.077** | **5.588** | **<0.001** | **-0.579** | **-0.279** |
|  |  | Rainfall | 0.142 | 0.180 | 0.180 | 0.790 | 0.429 | -0.210 | 0.494 |
|  |  | Tree characteristics | 0.008 | 0.028 | 0.028 | 0.289 | 0.772 | -0.047 | 0.063 |
|  |  | Colony present * Rainfall | 0.007 | 0.033 | 0.034 | 0.207 | 0.836 | -0.059 | 0.072 |
|  |  |  |  |  |  |  |  |  |  |
| b) Species richness | 2 | **Colony present** | **-0.203** | **0.036** | **0.036** | **5.592** | **<0.001** | **-0.275** | **-0.132** |
|  |  | Rainfall | 0.089 | 0.064 | 0.064 | 1.385 | 0.166 | -0.037 | 0.214 |
|  |  | Tree characteristics | 0.013 | 0.022 | 0.022 | 0.602 | 0.547 | -0.030 | 0.057 |
|  |  | **Colony present * Rainfall** | **0.106** | **0.036** | **0.036** | **2.917** | **0.004** | **0.035** | **0.178** |
|  |  |  |  |  |  |  |  |  |  |
| c) Shannon diversity | 5 | **Colony present** | **-0.109** | **0.033** | **0.033** | **3.292** | **<0.001** | **-0.173** | **-0.044** |
|  |  | Rainfall | 0.057 | 0.054 | 0.054 | 1.044 | 0.297 | -0.049 | -0.164 |
|  |  | Tree characteristics | 0.009 | 0.017 | 0.017 | 0.545 | 0.586 | -0.024 | 0.043 |
|  |  | **Colony present * Rainfall** | **0.084** | **0.033** | **0.033** | **2.571** | **0.010** | **0.020** | **0.145** |

**TABLE S5**. Top models explaining variation in the number of terrestrial invertebrates using NDVI as the stress component: number of individuals (a), species richness (b) and diversity captured at colony and non-colony trees. Global model: Colony present (yes/no) * NDVI + tree characteristics (PCA component one). Random terms: individual tree ID nested within colony ID. In total, 128 trees were monitored (64 colony trees, paired with 64 non-colony trees), each having six pitfall traps placed below. Of which, 320 pairs (n = 640) were without interference.

| Model | df | logLik | AICc | ΔAICc | Model weight |
| --- | --- | --- | --- | --- | --- |
| 1. Number of individuals |  |  |  |  |  |
| Colony present | 5 | -2012.37 | 4034.83 | 0.00 | 0.408 |
| Colony present + tree characteristics | 6 | -2012.16 | 4036.45 | 1.62 | 0.182 |
|  |  |  |  |  |  |
| 1. Species richness |  |  |  |  |  |
| Colony | 6 | -472.04 | 956.21 | 0.00 | 0.316 |
| Colony + tree characteristics | 7 | -471.53 | 957.23 | 1.02 | 0.189 |
| Colony * NDVI | 8 | -470.53 | 957.28 | 1.07 | 0.185 |
| Colony * NDVI + tree characteristics | 9 | -469.90 | 958.09 | 1.88 | 0.123 |
| Colony + NDVI | 7 | -472.01 | 958.20 | 1.99 | 0.117 |
|  |  |  |  |  |  |
| 1. Species diversity |  |  |  |  |  |
| Colony present | 5 | -373.44 | 756.98 | 0.00 | 0.306 |
| Colony present * NDVI | 7 | -371.76 | 757.69 | 0.71 | 0.214 |
| Colony present + tree characteristics | 6 | -373.06 | 758.26 | 1.28 | 0.161 |
| Colony present * NDVI + tree characteristics | 8 | -371.28 | 758.80 | 1.82 | 0.123 |
| Colony present + NDVI | 6 | -373.34 | 758.82 | 1.84 | 0.121 |

**TABLE S6.** Factors affecting terrestrial invertebrate abundance using NDVI as the stress component. Model estimates are averaged across two competing models for the number of daily individuals (a), four competing models for species richness (b) and five competing models for Shannon diversity (c), within ∆AICc = 2.0.

| Model | # Competing models | Predictor | Estimate | Std. error | Adjusted SE | Z value | *p* value | 2.5% CI | 97.5% CI |
| --- | --- | --- | --- | --- | --- | --- | --- | --- | --- |
| a) Number of individuals | 2 | **Colony present** | **-0.428** | **0.077** | **0.077** | **5.578** | **<0.0001** | **1.871** | **2.633** |
|  |  | Tree characteristics | 0.009 | 0.030 | 0.030 | 0.319 | 0.749 | -0.049 | 0.068 |
|  |  |  |  |  |  |  |  |  |  |
| b) Species richness | 5 | **Colony present** | **-0.194** | **0.037** | **0.037** | **5.277** | **<0.001** | **-0.265** | **-0.122** |
|  |  | NDVI | **-0.001** | **0.057** | **0.057** | **0.010** | **0.002** | **-0.112** | **0.111** |
|  |  | Tree characteristics | 0.009 | 0.188 | 0.188 | 0.462 | 0.644 | -0.028 | 0.045 |
|  |  | Colony present * NDVI | 0.020 | 0.035 | 0.350 | 0.576 | 0.564 | -0.484 | 0.088 |
|  |  |  |  |  |  |  |  |  |  |
| c) Species diversity | 5 | **Colony present** | **-0.108** | **0.033** | **0.033** | **3.258** | **0.001** | **-0.172** | **-0.043** |
|  |  | NDVI | 0.003 | 0.044 | 0.044 | 0.067 | 0.947 | -0.084 | 0.090 |
|  |  | Tree characteristics | 0.006 | 0.014 | 0.014 | 0.402 | 0.688 | -0.222 | 0.034 |
|  |  | Colony present * NDVI | 0.021 | 0.035 | 0.035 | 0.621 | 0.534 | 0.046 | 0.090 |

**TABLE S7**. Top models explaining variation in the number of aerial invertebrates using rainfall as the stress component: number of individuals (a), species richness (b) and diversity captured at colony and non-colony trees. Global model: Colony present (yes/no) * NDVI + tree characteristics (PCA component one). Random terms: individual tree ID nested within colony ID. In total, 128 trees were monitored (64 colony trees, paired with 64 non-colony trees), each having six pitfall traps placed below. Of which, 320 pairs (n = 640) were without interference.

| Model | df | logLik | AICc | ΔAICc | Model weight |
| --- | --- | --- | --- | --- | --- |
| 1. Number of individuals |  |  |  |  |  |
| Colony present + Rainfall + tree characteristics + | 7 | -417.21 | 849.43 | 0.00 | 0.377 |
| Colony present + Rainfall | 6 | -418.56 | 849.87 | 0.44 | 0.303 |
| Colony present * Rainfall + tree characteristics | 8 | -416.89 | 851.11 | 1.67 | 0.164 |
| Colony present * Rainfall | 7 | -418.15 | 851.32 | 1.88 | 0.147 |
|  |  |  |  |  |  |
| 1. Species richness |  |  |  |  |  |
| Colony present + Rainfall + tree characteristics | 7 | -78.67 | 172.37 | 0.00 | 0.512 |
| Colony present + Rainfall | 6 | -80.68 | 174.12 | 1.74 | 0.214 |
|  |  |  |  |  |  |
| 1. Species diversity |  |  |  |  |  |
| Colony present + Rainfall + tree characteristics | 8 | -68.15 | 151.33 | 0.00 | 0.360 |
| Colony present + Rainfall | 7 | -69.42 | 151.57 | 0.27 | 0.314 |

**TABLE S8.** Factors affecting aerial invertebrate abundance using rainfall as the stress component. Model estimates are averaged across four competing models for the number of daily individuals (a), two competing models for species richness (b) and two competing models for Shannon diversity (c), within ∆AICc = 2.0.

| Model | # Competing models | Predictor | Estimate | Std. error | Adjusted SE | Z value | *p* value | 2.5% CI | 97.5% CI |
| --- | --- | --- | --- | --- | --- | --- | --- | --- | --- |
| a) Number of individuals | 4 | **Colony present** | **-0.297** | **0.090** | **0.091** | **3.274** | **0.001** | **-0.475** | **-0.119** |
|  |  | **Rainfall** | **0.466** | **0.088** | **0.089** | **5.218** | **<0.001** | **0.291** | **0.640** |
|  |  | Tree characteristics | 0.051 | 0.063 | 0.064 | 0.794 | 0.426 | -0.074 | 0.176 |
|  |  | Colony present * Rainfall | -0.026 | 0.066 | 0.067 | 0.385 | 0.700 | -0.156 | 0.105 |
|  |  |  |  |  |  |  |  |  |  |
| b) Species richness | 2 | **Colony present** | **-0.270** | **0.072** | **0.073** | **3.702** | **<0.001** | **-0.413** | **-0.127** |
|  |  | **Rainfall** | **0.266** | **0.071** | **0.071** | **3.724** | **<0.001** | **0.126** | **0.406** |
|  |  | Tree characteristics | 0.064 | 0.056 | 0.056 | 1.380 | 0.255 | -0.046 | 0.174 |
|  |  |  |  |  |  |  |  |  |  |
| c) Shannon diversity | 2 | **Colony present** | **-0.263** | **0.068** | **0.068** | **3.897** | **0.001** | **-0.397** | **-0.129** |
|  |  | **Rainfall** | **0.187** | **0.063** | **0.064** | **2.910** | **0.004** | **0.061** | **0.316** |
|  |  | Tree characteristics | 0.035 | 0.045 | 0.045 | 0.786 | 0.432 | -0.053 | 0.124 |

**TABLE S9**. Top models explaining variation in the number of aerial invertebrates using NDVI as the stress component: number of individuals (a), species richness (b) and diversity captured at colony and non-colony trees. Global model: Colony present (yes/no) * NDVI + tree characteristics (PCA component one). Random terms: individual tree ID nested within colony ID. In total, 128 trees were monitored (64 colony trees, paired with 64 non-colony trees), each having six pitfall traps placed below. Of which, 320 pairs (n = 640) were without interference.

| Model | df | logLik | AICc | ΔAICc | Model weight |
| --- | --- | --- | --- | --- | --- |
| 1. Number of individuals |  |  |  |  |  |
| Colony present + NDVI + tree characteristics | 7 | -418.31 | 851.64 | 0.00 | 0.364 |
| Colony present + NDVI | 6 | -419.48 | 851.71 | 0.07 | 0.351 |
| Colony present * NDVI + tree characteristics | 8 | -418.14 | 853.61 | 1.96 | 0.136 |
|  |  |  |  |  |  |
| 1. Species richness |  |  |  |  |  |
| Colony present + NDVI + tree characteristics | 7 | -80.41 | 175.84 | 0.00 | 0.324 |
| Colony present * NDVI + tree characteristics | 8 | -79.30 | 175.92 | 0.07 | 0.312 |
| Colony + NDVI | 6 | -82.39 | 177.54 | 1/70 | 0.138 |
|  |  |  |  |  |  |
| 1. Species diversity |  |  |  |  |  |
| Colony present * NDVI + tree characteristics | 8 | -68.18 | 153.68 | 0.00 | 0.233 |
| Colony present + NDVI + tree characteristics | 7 | -69.44 | 153.90 | 0.21 | 0.209 |
| Colony present + NDVI | 6 | -70.73 | 154.21 | 0.52 | 0.180 |
| Colony present * NDVI | 7 | -69.73 | 154.46 | 0.77 | 0.158 |
| Colony present + tree characteristics | 6 | -71.07 | 154.89 | 1.21 | 0.128 |
| Colony present | 5 | -72.54 | 155.60 | 1.92 | 0.089 |

**TABLE S10.** Factors affecting aerial invertebrate abundance using NDVI as the stress component. Model estimates are averaged across three competing models for the number of daily individuals (a), three competing models for species richness (b) and six competing models for Shannon diversity (c), within ∆AICc = 2.0.

| Model | # Competing models | Predictor | Estimate | Std. error | Adjusted SE | Z value | *p* value | 2.5% CI | 97.5% CI |
| --- | --- | --- | --- | --- | --- | --- | --- | --- | --- |
| a) Terrestrial animal  events | 3 | **Colony present** | **-0.299** | **0.089** | **0.089** | **3.335** | **<0.001** | **-0.474** | **-0.123** |
|  |  | **NDVI** | **0.410** | **0.085** | **0.086** | **4.747** | **<0.0001** | **0.241** | **0.579** |
|  |  | Tree characteristics | 0.052 | 0.063 | 0.063 | 0.823 | 0.410 | -0.072 | 0.176 |
|  |  | Colony present * NDVI | 0.007 | 0.035 | 0.035 | 0.204 | 0.838 | -0.062 | 0.076 |
|  |  |  |  |  |  |  |  |  |  |
| b) Species richness | 3 | **Colony present** | **-0.358** | **0.156** | **0.157** | **2.280** | **0.023** | **-0.665** | **-0.050** |
|  |  | **NDVI** | **1.802** | **0.855** | **0.864** | **2.085** | **0.038** | **0.108** | **3.495** |
|  |  | Tree characteristics | 0.078 | 0.055 | 0.055 | 1.409 | .0.159 | -0.030 | 0.186 |
|  |  | Colony present * NDVI | 0.391 | 0.628 | 0.631 | 0.621 | 0.535 | -0.844 | 1.627 |
|  |  |  |  |  |  |  |  |  |  |
| c) Species diversity | 6 | **Colony present** | **-0.343** | **0.146** | **0.147** | **2.334** | **0.020** | **-0.631** | **-0.055** |
|  |  | NDVI | 0.882 | 0.814 | 0.820 | 1.076 | 0.282 | -0.724 | 2.489 |
|  |  | Tree characteristics | 0.040 | 0.047 | 0.047 | 0.854 | 0,393 | -0.052 | 0.133 |
|  |  | Colony present * NDVI | 0.367 | 0.596 | 0.598 | 0.613 | 0.540 | -0.806 | 1.540 |

**TABLE S11.** Top models explaining variation in the number of reptiles at colony and non-colony trees using NDVI as the stress component. Global model: Colony present (yes/no) * NDVI + tree characteristics (PCA component one). Random terms: individual tree ID nested within colony ID. N = 128 Trees.

| Model | df | logLik | AICc | ΔAICc | Model weight |
| --- | --- | --- | --- | --- | --- |
| Number of Reptiles |  |  |  |  |  |
| Colony present | 4 | -124.46 | 257.24 | 0.00 | 0.266 |
| Colony present + NDVI | 5 | -124.31 | 259.10 | 1.86 | 0.105 |
| Colony present * NDVI | 6 | -123.24 | 259.18 | 1.95 | 0.101 |

**TABLE S12.** Factors affecting reptile abundance using NDVI as the stress component. Model estimates are averaged across three competing models within ∆AICc = 2.0

| Model | # Competing models | Predictor | Estimate | Std. error | Adjusted SE | Z value | *p* value | 2.5% CI | 97.5% CI |
| --- | --- | --- | --- | --- | --- | --- | --- | --- | --- |
| a) Number of reptiles  trap events | 3 | **Colony present (Yes/No)** | **-0.522** | **0.233** | **0.236** | **0.213** | **0.027** | **-0.985** | **-0.060** |
|  |  | NDVI | -0.056 | 0.285 | 0.287 | 0.194 | 0.846 | -0.620 | 0.508 |
|  |  | Colony present * NDVI | -0.124 | 0.305 | 0.306 | 0.406 | 0.685 | -0.724 | 0.476 |

**TABLE S13.** Top models explaining variation in the number of reptiles at colony and non-colony trees using rainfall as the stress component. Global model: Colony present (yes/no) * Rainfall + tree characteristics (PCA component one). Random terms: individual tree ID nested within colony ID. N = 128 Trees.

| Model | df | logLik | AICc | ΔAICc | Model weight |
| --- | --- | --- | --- | --- | --- |
| Number of Reptiles |  |  |  |  |  |
| Colony present | 4 | -124.46 | 257.24 | 0.00 | 0.198 |
| Colony present * rainfall | 6 | -122.38 | 257.45 | 1.20 | 0.179 |
| Colony present + rainfall | 5 | -123.96 | 258.42 | 1.18 | 0.108 |

**TABLE S14.** Factors affecting reptile abundance using rainfall as the stress component. Model estimates are averaged across three competing models within ∆AICc = 2.0

| Model | # Competing models | Predictor | Estimate | Std. error | Adjusted SE | Z value | *p* value | 2.5% CI | 97.5% CI |
| --- | --- | --- | --- | --- | --- | --- | --- | --- | --- |
| a) Number of reptiles  trap events | 3 | Colony present (Yes/No) | -0.467 | 0.242 | 0.245 | 1.901 | 0.056. | -0.946 | 0.013 |
|  |  | Rainfall | 0.333 | 0.443 | 0.445 | 0.747 | 0.455 | -0.540 | 1.206 |
|  |  | Colony present * rainfall | -0.213 | 0.346 | 0.348 | 0.615 | 0.539 | -0.894 | 0.467 |

**TABLE S15.** Top models explaining avian abundance and diversity using rainfall as the stress component: number of individuals (a), species richness (b) and species diversity at colony and non-colony trees. Global model: Colony present (yes/no) * NDVI + tree characteristics (PCA component one). Random terms: individual tree ID nested within colony ID. N = 124 point counts.

| Model | df | logLik | AICc | ΔAICc | Model weight |
| --- | --- | --- | --- | --- | --- |
| 1. Number of individual birds |  |  |  |  |  |
| Colony present * rainfall | 7 | -156.758 | 328.450 | 0.00 | 0.683 |
| Colony present * rainfall + tree characteristics | 8 | -156.574 | 330.359 | 1.91 | 0.263 |
|  |  |  |  |  |  |
| 1. Species richness |  |  |  |  |  |
| Colony present * rainfall | 6 | -131.983 | 276.660 | 0.00 | 0.540 |
|  |  |  |  |  |  |
| 1. Species diversity |  |  |  |  |  |
| Intercept only | 4 | -40.691 | 89.707 | 0.00 | 0.266 |
| Rainfall | 5 | -40.048 | 90.588 | 0.88 | 0.171 |
| Colony | 5 | -40.148 | 91.328 | 1.62 | 0.118 |
| Tree characteristics | 5 | -50.504 | 91.501 | 1.79 | 0.108 |

**TABLE S16.** Factors affecting avian abundance using rainfall as the stress component. Model estimates are averaged across two competing models (within ∆AICc = 2.0) for the total number of individuals (a), one for species richness (b), and four competing models for Shannon species diversity (c).

| Model | # Competing models | Predictor | Estimate | Std. error | Adjusted SE | Z value | *p* value | 2.5% CI | 97.5% CI |
| --- | --- | --- | --- | --- | --- | --- | --- | --- | --- |
| a) Number of individuals | 2 | **Colony present (Yes/No)** | **-0.654** | **0.260** | **0.262** | **2.460** | **0.013** | **-1.160** | **-0.131** |
|  |  | Rainfall | 0.094 | 0.200 | 0.202 | 0.466 | 0.641 | -0.301 | 0.489 |
|  |  | Tree characteristics (PCA) | 0.049 | 0.144 | 0.145 | 0.266 | 0.790 | -0.246 | 0.324 |
|  |  | **Colony present (Yes/No) * Rainfall** | **0.759** | **0.243** | **0.245** | **3.101** | **0.002** | **0.279** | **1.239** |
|  |  |  |  |  |  |  |  |  |  |
| b) Species richness | 1 | Colony present (Yes/No) | -0.431 | 0.239 |  | -1.803 | 0.071 | -0.899 | 0.037 |
|  |  | Rainfall | 0.053 | 0.214 |  | 0.242 | 0.809 | -0.368 | 0.471 |
|  |  | **Colony presence (Yes/No) * Rainfall** | **0.636** | **0.239** |  | **2.660** | **0.006** | **0.167** | **1.105** |
|  |  |  |  |  |  |  |  |  |  |
| c) Shannon diversity | 4 | Colony present (Yes/No) | 0.007 | 0.029 | 0.029 | 0.257 | 0.797 | -0.049 | 0.064 |
|  |  | Rainfall | 0.014 | 0.032 | 0.032 | 0.418 | 0.676 | -0.050 | 0.077 |
|  |  | Tree characteristics (PCA) | 0.003 | 0.014 | 0.014 | 0.214 | 0.831 | -0.024 | 0.030 |

**TABLE S17.** Top models explaining avian abundance and diversity using NDVI as the stress component: number of individuals (a), species richness (b) and species diversity at colony and non-colony trees. Global model: Colony present (yes/no) * NDVI + tree characteristics (PCA component one). Random terms: individual tree ID nested within colony ID. N = 124 point counts.

| Model | df | logLik | AICc | ΔAICc | Model weight |
| --- | --- | --- | --- | --- | --- |
| 1. Number of individual birds |  |  |  |  |  |
| Colony present * NDVI | 7 | -159.57 | 334.01 | 0.00 | 0.339 |
| Colony present * NDVI + tree characteristics | 6 | -159.29 | 335.78 | 1.69 | 0.145 |
| Colony present + NDVI | 8 | -161.69 | 336.07 | 1.98 | 0.126 |
|  |  |  |  |  |  |
| 1. Species richness |  |  |  |  |  |
| Colony present * NDVI | 6 | -133.032 | 278.756 | 0.00 | 0.330 |
| NDVI | 4 | -136.047 | 280.419 | 1.66 | 0.144 |
| Colony present * NDVI + tree characteristics | 7 | -132.908 | 280.750 | 1.99 | 0.122 |
|  |  |  |  |  |  |
| 1. Species diversity |  |  |  |  |  |
| Colony * NDVI | 7 | -37.145 | 89.223 | 0.00 | 0.237 |
| Colony | 4 | -40.691 | 89.707 | 0.48 | 0.186 |
| Colony + NDVI | 5 | -39.816 | 0.123 | 0.90 | 0.151 |

**TABLE S18.** Factors affecting avian abundance using NDVI as the stress component. Model estimates are averaged across three competing models (within ∆AICc = 2.0) for the total number of individuals (a), three for species richness (b), and three competing models for Shannon species diversity (c).

| Model | # Competing models | Predictor | Estimate | Std. error | Adjusted SE | Z value | *p* value | 2.5% CI | 97.5% CI |
| --- | --- | --- | --- | --- | --- | --- | --- | --- | --- |
| a) Number of individuals | 3 | Colony present (Yes/No) | -0.470 | 0.250 | 0.253 | 1.857 | 0.063 | -0.965 | 0.026 |
|  |  | NDVI | 0.215 | 0.224 | 0.226 | 0.948 | 0.343 | -0.216 | 0.659 |
|  |  | Tree characteristics (PCA) | 0.038 | 0.134 | 0.134 | 0.282 | 0.778 | -0.226 | 0.788 |
|  |  | Colony present * NDVI | 0.317 | 0.239 | 0.240 | 1.321 | 0.187 | -0.154 | 0.788 |
|  |  |  |  |  |  |  |  |  |  |
| b) Species richness | 3 | Colony present (Yes/No) | -0.256 | 0.243 | 0.245 | 1.046 | 0.295 | -0.735 | 0.223 |
|  |  | NDVI | 0.159 | 0.230 | 0.232 | 0.683 | 0.494 | -0.297 | 0.614 |
|  |  | Tree characteristics (PCA) | 0.018 | 0.098 | 0.098 | 0.190 | 0.849 | -0.174 | 0.212 |
|  |  | Colony presence * NDVI | 0.318 | 0.245 | 0.247 | 1.288 | 0.198 | 0.166 | 0.801 |
|  |  |  |  |  |  |  |  |  |  |
| c) Shannon diversity | 3 | Colony present (Yes/No) | 0.042 | 0.056 | 0.056 | 0.746 | 0.456 | -0.068 | 0.152 |
|  |  | NDVA | 0.011 | 0.053 | 0.053 | 0.209 | 0.835 | -0.093 | 0.116 |
|  |  | Colony present * NDVI | 0.075 | 0.074 | 0.074 | 1.016 | 0.310 | -0.070 | 0.221 |

**TABLE S19** Results from the generalised linear mixed models investigating roosting birds, using Rainfall as the stress component, at colony trees only in relation to colony size, rainfall. All model terms, error distributions, transformation and zero-inflations are listed in Table S2.

| Response variables | Explanatory variables | Estimate | + SE | χ^2^ | *P* |
| --- | --- | --- | --- | --- | --- |
| a) Colony occupancy | **Colony size** | **1.386** | **0.490** | **8.008** | **0.005** |
| (yes/no) n = 64 | **Rainfall** | **0.807** | **0.349** | **5.336** | **0.021** |
|  |  |  |  |  |  |
| b) Roosting bird abundance | Colony size | 0.332 | 0.199 | 2.786 | 0.091 |
| n=64 | Rainfall | -0.098 | 0.195 | 0.251 | 0.616 |
|  |  |  |  |  |  |
| c) Species richness | **Colony size** | **0.242** | **0.128** | **3.890** | **0.049** |
| n=64 | Rainfall | 0.147 | 0.162 | 0.813 | 0.367 |
|  |  |  |  |  |  |
| d) Species diversity | Colony size | 0.061 | 0.041 | 1.494 | 0.135 |
| n=64 | Rainfall | 0.033 | 0.051 | 0.648 | 0.516 |

**TABLE S20** Results from the generalised linear mixed models, using NDVI as the stress component, investigating roosting birds at colony trees only in relation to colony size and NDVI. All model terms, error distributions, transformation and zero-inflations are listed in Table S2.

| Response variables | Explanatory variables | Estimate | + SE | χ^2^ | *P* |
| --- | --- | --- | --- | --- | --- |
| a) Colony occupancy | **Colony size** | **1.572** | **0.494** | **10.1199** | **0.001** |
| (yes/no) n = 64 | **NDVI** | **0.659** | **0.317** | **4.329** | **0.038** |
|  |  |  |  |  |  |
| b) Roosting bird abundance | Colony size | 0.005 | 0.004 | 1.787 | 0.181 |
| n=64 | NDVI | -0.201 | 0.171 | 1.511 | 0.212 |
|  |  |  |  |  |  |
| c) Species richness | **Colony size** | **0.005** | **0.002** | **4.715** | **0.030** |
| n=64 | NDVI | 0.092 | 0.155 | 0.353 | 0.552 |
|  |  |  |  |  |  |
| d) Species diversity | Colony size | 0.001 | 0.001 | 2.373 | 0.124 |
| n=64 | NDVI | 0.001 | 0.051 | 0.008 | 0.978 |

**TABLE S21.** Top models explaining variation in the number of camera trap events using rainfall as the stress component (a), the event duration (b), species richness (c) and Shannon diversity (d) at colony and non-colony trees. Global model: Colony present (yes/no) * NDVI + tree characteristics (PCA component one). Random terms: individual tree ID nested within colony ID. N = 128 Trees.

| Model | df | logLik | AICc | ΔAICc | Model weight |
| --- | --- | --- | --- | --- | --- |
| 1. Number of camera trap events |  |  |  |  |  |
| Colony present + rainfall | 6 | 248.03 | 507.76 | 0.00 | 0.470 |
| Colony present * rainfall | 7 | 247.65 | 510.24 | 1.48 | 0.224 |
| Colony present + rainfall + tree characteristics | 7 | 247.82 | 510.56 | 1.81 | 0.190 |
|  |  |  |  |  |  |
| 1. Duration of event |  |  |  |  |  |
| Colony present + rainfall | 6 | -358.99 | 730.68 | 0.00 | 0.400 |
| Colony present * rainfall | 7 | -358.12 | 731.17 | 0.49 | 0.312 |
|  |  |  |  |  |  |
| 1. Species richness |  |  |  |  |  |
| Rainfall | 5 | -226.70 | 463.90 | 0.00 | 0.333 |
| Colony present + rainfall | 6 | -226.03 | 464.75 | 0.85 | 0.218 |
| Colony present * rainfall | 7 | -225.01 | 464.94 | 1.04 | 0.198 |
|  |  |  |  |  |  |
| 1. Species diversity |  |  |  |  |  |
| Colony present + rainfall | 6 | -67.410 | 147.51 | 0.00 | 0.376 |
| Colony present + rainfall + tree characteristics | 7 | -66.719 | 148.37 | 0.86 | 0.245 |
| Colony present * rainfall | 7 | -66.774 | 148.48 | 0.97 | 0.232 |

**TABLE S22.** Factors affecting camera trap events abundance using rainfall as the stress component. Model estimates are averaged across three competing models within ∆AICc = 2.0 for the number of daily animal events (a), two for event duration (b), three for species richness (c) and three for species diversity (d).

| Model | # Competing models | Predictor | Estimate | Std. error | Adjusted SE | Z value | *p* value | 2.5% CI | 97.5% CI |
| --- | --- | --- | --- | --- | --- | --- | --- | --- | --- |
| a) Number of camera trap events | 3 | **Colony present (Yes/No)** | **-0.657** | **0.216** | **0.219** | **3.010** | **0.027** | **-1.086** | **-0.229** |
|  |  | **Rainfall** | **0.557** | **0.122** | **0.124** | **4.525** | **<0.001** | **0.315** | **0.798** |
|  |  | Tree characteristics (PCA) | 0.016 | 0.062 | 0.062 | 0.263 | 0.792 | -0.106 | 0.139 |
|  |  | Colony present * Rainfall | 0.048 | 0.138 | 0.139 | 0.347 | 0.729 | -0.224 | 0.320 |
|  |  |  |  |  |  |  |  |  |  |
| b) Event duration | 2 | **Colony present** | **-1.147** | **0.368** | **0.371** | **3.088** | **0.002** | **-1.875** | **0.419** |
|  |  | **Rainfall** | **0.687** | **0.287** | **0.290** | **2.370** | **0.018** | **0.119** | **1.254** |
|  |  | Colony present * Rainfall | 0.225 | 0.365 | 0.366 | 0.615 | 0.539 | -0.493 | 0.944 |
|  |  |  |  |  |  |  |  |  |  |
| c) Species richness | 3 | Colony present | -1.053 | 0.142 | 0.142 | 0.740 | 0.460 | -0.384 | 0.174 |
|  |  | Rainfall | 0.391 | 0.101 | 0.102 | 3.849 | <0.001 | 0.192 | 0.590 |
|  |  | Colony present * Rainfall | 0.053 | 0.114 | 0.115 | 0.464 | 0.643 | -0.172 | 0.278 |
|  |  |  |  |  |  |  |  |  |  |
| d) Species diversity | 2 | **Colony present (Yes/No)** | **-0.251** | **0.072** | **0.073** | **3.437** | **0.001** | **-0.394** | **-0.108** |
|  |  | **Rainfall** | **0.153** | **0.036** | **0.037** | **4.148** | **<0.001** | **0.081** | **0.225** |
|  |  | Tree characteristics (PCA) | 0.017 | 0.031 | 0.031 | 0.543 | 0.567 | -0.044 | 0.078 |

**TABLE S23.** Top models explaining variation in the number of camera trap events, using NDVI as the stress component (a), the event duration (b), species richness (c) and Shannon diversity (d) at colony and non-colony trees. Global model: Colony present (yes/no) * NDVI + tree characteristics (PCA component one). Random terms: individual tree ID nested within colony ID. N = 128 Trees.

| Model | df | logLik | AICc | ΔAICc | Model weight |
| --- | --- | --- | --- | --- | --- |
| 1. Number of camera trap events |  |  |  |  |  |
| Colony present + NDVI | 6 | -251.97 | 516.28 | 0.00 | 0.47 |
| Colony present + NDVI + tree characteristics | 7 | -251.61 | 518.16 | 0.00 | 0.18 |
|  |  |  |  |  |  |
| 1. Duration of event |  |  |  |  |  |
| Colony present + NDVI | 6 | -361.70 | 736.10 | 0.00 | 0.45 |
| Colony present * NDVI | 7 | -361.58 | 738.10 | 1.99 | 0.16 |
|  |  |  |  |  |  |
| 1. Species richness |  |  |  |  |  |
| NDVI | 5 | -231.85 | 474.19 | 0.00 | 0.233 |
| Intercept only | 4 | -231.16 | 474.65 | 0.46 | 0.185 |
| Colony present + NDVI | 6 | -231.17 | 475.04 | 0.84 | 0.153 |
| Colony present | 5 | -232.50 | 475.50 | 1.30 | 0.121 |
|  |  |  |  |  |  |
| 1. Species diversity |  |  |  |  |  |
| Colony present + NDVI | 6 | -70.14 | 152.97 | 0.00 | 0.304 |
| Colony present * NDVI | 7 | -69.11 | 153.16 | 0.19 | 0.277 |
| Colony present + NDVI + tree characteristics | 7 | -69.54 | 154.01 | 1.04 | 0.181 |
| Colony present * NDVI + tree characteristics | 8 | -68.62 | 154.45 | 1.48 | 0.145 |

**TABLE S24.** Factors affecting camera trap events abundance using NDVI as the stress component. Model estimates are averaged across two competing models within ∆AICc = 2.0 for the number of daily animal events (a), two for event duration (b), four for species richness (c) and three for species diversity (d).

| Model | # Competing models | Predictor | Estimate | Std. error | Adjusted SE | Z value | *p* value | 2.5% CI | 97.5% CI |
| --- | --- | --- | --- | --- | --- | --- | --- | --- | --- |
| a) Number of camera  trap events | 2 | **Colony present (Yes/No)** | **-0.644** | **0.209** | **0.211** | **3.05** | **0.002** | **-1.058** | **-0.230** |
|  |  | **NDVI** | **0.458** | **0.172** | **0.174** | **2.64** | **0.008** | **0.118** | **0.799** |
|  |  | Tree characteristics (PCA) | 0.020 | 0.072 | 0.072 | 0.28 | 0.780 | -0.121 | 0.161 |
|  |  |  |  |  |  |  |  |  |  |
| b) Event duration | 2 | **Colony present** | **-1.097** | **0.373** | **0.377** | **2.912** | **0.004** | **-2.311** | **-0.078** |
|  |  | **NDVI** | **5.053** | **2.341** | **2.364** | **2.138** | **0.033** | **0.046** | **1.058** |
|  |  | Colony present * NDVI | 0.050 | 0.217 | 0.219 | 0.230 | 0.818 | -0.378 | 0.479 |
|  |  |  |  |  |  |  |  |  |  |
| c) Species richness | 4 | Colony present | -0.062 | 0.114 | 0.115 | 0.538 | 0.591 | -0.286 | -0.163 |
|  |  | NDVI | 0.127 | 0.147 | 0.148 | 0.860 | 0.390 | -0.163 | 0.417 |
|  |  |  |  |  |  |  |  |  |  |
| d) Species diversity | 3 | **Colony present (Yes/No)** | **-0.250** | **0.072** | **0.073** | **3.450** | **<0.001** | **-0.393** | **-0.108** |
|  |  | **NDVI** | **0.146** | **0.055** | **0.055** | **2.637** | **0.008** | **0.038** | **0.255** |
|  |  | Tree characteristics (PCA) | 0.015 | 0.030 | 0.030 | 0.479 | 0.632 | -0.045 | 0.074 |
|  |  | Colony present * NDVI | 0.014 | 0.030 | 0.030 | 0.479 | 0.632 | -0.045 | 0.074 |
